# Inhibition of Aberrant Activated Fibroblast‐Like Synoviocytes in Rheumatoid Arthritis by *Leishmania* Peptide via the Regulation of Fatty Acid Synthesis Metabolism

**DOI:** 10.1002/advs.202409154

**Published:** 2025-03-24

**Authors:** Jianling Su, Xuemei Fan, Yaoyao Zou, Guangtao Fu, Shiqi Feng, Xiaoxue Wang, Yongmei Yu, Lin Li, Zhenhua Bian, Rongrong Huang, Linmang Qin, Jiping Chen, Qin Zeng, Kai Yan, Caiyue Gao, Zhexiong Lian, Xin Li, Yang Li

**Affiliations:** ^1^ Guangdong Cardiovascular Institute Guangdong Provincial People's Hospital Guangdong Academy of Medical Sciences Guangzhou 510000 China; ^2^ Department of Rheumatology and Immunology Guangdong Provincial People's Hospital (Guangdong Academy of Medical Sciences) Southern Medical University Guangzhou 510000 China; ^3^ Department of Rheumatology Zibo Central Hospital Zibo Shandong 255036 China; ^4^ Department of Orthopedics Guangdong Provincial People's Hospital (Guangdong Academy of Medical Sciences) Southern Medical University Guangzhou 510000 China; ^5^ The First Affiliated Hospital of Shenzhen University Shenzhen Second People's Hospital Shenzhen University Shenzhen 518035 China; ^6^ Department of Rheumatology and Immunology 2nd Affiliated Hospital of Harbin Medical University Harbin 150001 China; ^7^ School of Biomedical Sciences and Engineering South China University of Technology Guangzhou International Campus Guangzhou 511442 China; ^8^ Department of Pharmacy Guangdong Provincial People's Hospital (Guangdong Academy of Medical Sciences) Southern Medical University Guangzhou 510080 China; ^9^ Medical Research Institute Guangdong Provincial People's Hospital (Guangdong Academy of Medical Sciences) Southern Medical University Guangzhou 510080 China; ^10^ Guangdong Provincial People's Hospital (Guangdong Academy of Medical Sciences) Southern Medical University Guangzhou 510000 China

**Keywords:** FASN, fatty acid synthesis metabolism, fibroblast‐like synoviocytes, *Leishmania* receptors for activated C kinase, rheumatoid arthritis

## Abstract

The *Leishmania* homolog of receptors for activated C kinase (LACK) protein is derived from *Leishmania* parasites *L. major*. The polypeptide LACK_156–173_ has been shown to confer protection against murine autoimmune arthritis. Fibroblast‐like synoviocytes (FLSs) play a pivotal role in the synovial invasion and joint destruction observed in rheumatoid arthritis (RA). The study reveals that LACK_156‐173_ can inhibit the aggressive phenotype of RA‐FLSs by restoring dysregulated fatty acid synthesis metabolism. In RA‐FLSs, overexpression of fatty acid synthase (FASN) leads to excessive fatty acid accumulation, which in turn promotes mitochondrial fragmentation by enhancing phosphorylation at the ser616 site of dynamin 1‐like protein (DRP1). This process increases reactive oxygen species (ROS) production and activates the PI3K/mTOR/NF‐κB pathway, thereby facilitating the transition of RA‐FLSs to an aggressive inflammatory and bone‐damaging phenotype. LACK_156‐173_ is internalized into the cytoplasm via CAPN2‐mediated endocytosis, where it directly binds to FASN and inhibits its activity. The findings suggest that targeting the restoration of fatty acid metabolism could potentially alleviate synovial invasion and joint damage in RA. LACK_156‐173_ may therefore hold therapeutic promise for RA patients.

## Graphical abstract

LACK_156‐173_ is internalized into RA‐FLSs via CAPN2‐mediated endocytosis and inhibits the aggressive phenotype of RA‐FLSs by restoring dysregulated fatty acid synthesis metabolism mediated by overexpressed FASN. The findings suggest that targeting the restoration of fatty acid metabolism could potentially alleviate synovial invasion and joint damage in RA. Therefore, LACK_156‐173_ may hold therapeutic promise for RA patients.

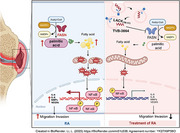

## 1 Introduction

Rheumatoid arthritis (RA) is a chronic autoimmune inflammatory disease affecting synovial joints, where the synovium becomes inflamed and forms pannus, leading to cartilage and bone destruction.^[^ ^1^ ^]^ According to the Global Burden of Disease (GBD) 2017 study, the global age‐standardized prevalence of RA has increased by 7.4%.^[^ ^2^ ^]^ T cell‐mediated adaptive immunity has been linked to the development of RA.^[^ ^3^ ^]^ Nevertheless, recent attention has shifted to fibroblast‐like synoviocytes (FLSs) in RA pathogenesis. FLSs are now recognized as key drivers of synovial inflammation and joint damage, rather than merely “passive responders”.^[^ ^1b^ ^]^ Expression of cytokines, matrix metalloproteinases (MMPs), and vascular endothelial growth factor (VEGF) by FLSs promotes cellular infiltration, pannus formation, and angiogenesis. This process involves the receptor activator of nuclear factor‐κB ligand (RANKL), which induces osteoclastogenesis, leading to osteoclastic bone destruction, and enzymatic cartilage degradation.^[^ ^1^, ^4^ ^]^ FLSs have emerged as a therapeutic target in RA to avoid side effects such as systemic immune suppression associated with many current RA treatments.^[^ ^5^ ^]^ However, therapeutic options that specifically and directly target RA‐FLSs in individuals with RA are still lacking.

Over the past 30 years, the “hygiene hypothesis” has suggested that reduced exposure to common organisms, such as helminths, may contribute to increased prevalence of allergic and autoimmune diseases.^[^ ^6^ ^]^ While the immunomodulatory effects of parasites and their excretory‐secretory products in modulating inflammatory diseases, including RA, are well‐documented, their specific impact on stromal cells and the processes of bone and cartilage destruction in these conditions remains inadequately studied. In our previous study, we discovered that the *Leishmania* homolog of receptors for activated C kinase (LACK) protein, particularly its 156–173 peptide segment (ICFSPSLEHPIVVSGSWD, LACK_156‐173_), mediated protection against murine autoimmune arthritis by regulating dendritic cell (DC) differentiation and promoting Th2 cell polarization.^[^ ^7^ ^]^ LACK_156–173_ is presented by MHC class II I‐A^d^ molecules and exerts therapeutic effects in BALB/c mice (MHC class II I‐A^d^), but not C57BL/6 (MHC class II I‐A^b^) and DBA/1 mice (MHC class II I‐A^q^). The effect of LACK_156‐173_ on human RA‐FLSs and cartilage destruction has yet to be elucidated. To investigate the therapeutic potential of LACK_156‐173_ in human RA, we aim to explore its mode of action beyond the restriction of MHC class II I‐A^d^ molecular. Additionally, we seek to determine whether LACK_156‐173_ exerts its effects through alternative mechanisms in human RA.

In the inflammatory microenvironment of RA joints, FLSs undergo reprogrammed cellular metabolism involving glucose,^[^ ^8^ ^]^ fatty acids,^[^ ^9^ ^]^ and lactate metabolism.^[^ ^10^ ^]^ Disordered metabolites contribute to the aggressive phenotype of RA‐FLSs.^[^ ^11^ ^]^ Alterations in lipid metabolism are observed in FLSs even in the absence of synovial inflammation, suggesting that these changes precede clinical disease manifestation and may drive disease pathogenesis.^[^ ^12^ ^]^ Fatty acid synthase (FASN) plays a pivotal role in the fatty acid synthesis pathway by catalyzing the condensation of acetyl‐CoA and malonyl‐CoA to produce palmitic acid (PA). Overexpression of FASN increases the synthesis of saturated fatty acids, which has been implicated in tumor progression.^[^ ^13^ ^]^ Conversely, downregulation of FASN inhibits the activation of the AKT/mTOR pathway and cell proliferation genes, thereby delaying disease progression in conditions such as liver cancer.^[^ ^14^ ^]^ Metabolic reprogramming in tumor‐associated fibroblasts, facilitated by FASN‐mediated fatty acid synthesis, promotes the invasion of colon tumor cells.^[^ ^15^ ^]^ Normally, FASN‐mediated fatty acid synthesis coordinates lipid storage and mitochondrial function.^[^ ^16^ ^]^ However, excessive fatty acid synthesis can lead to lipid accumulation, disrupting the mitochondrial dynamic equilibrium towards fission and generating numerous mitochondrial fragments.^[^ ^17^ ^]^ Notably, RA‐FLSs exhibit a fragmented mitochondrial morphology, accompanied by heightened mitochondrial division, which is associated with an upregulation of the mitochondrial division protein DRP1 (DNM1L). Inhibition of DNM1L activity or expression reduces mitochondrial division, suppresses reactive oxygen species (ROS) expression, and alleviates murine autoimmune arthritis.^[^ ^18^ ^]^ Here, we demonstrate that LACK_156‐173_ reduces the aggressiveness of RA‐FLSs and inhibits the de novo fatty acid synthesis pathway mediated by FASN.

Our objective was to elucidate how fatty acid synthesis metabolism drives RA‐FLSs toward a hyper‐responsive phenotype that exacerbates joint pathology and to explore how LACK_156‐173_ disrupts these processes to promote a functional phenotype that mitigates joint damage in RA. Specifically, we observed elevated expression of FASN in RA‐FLSs, which contributes to their invasiveness and inflammatory characteristics through excessive fatty acid accumulation and increased mitochondrial fragmentation. LACK_156‐173_ enters FLSs via receptor‐mediated endocytosis, directly interacting with FASN to inhibit its activity. Our findings suggest that microbial products can attenuate fibroblast invasiveness via mechanisms distinct from those involving immune cells and that restoring disrupted fatty acid synthesis metabolism can alleviate the aggressive behavior of RA‐FLSs. These insights propose a potential therapeutic strategy for preventing RA‐FLSs from perpetuating inflammation and bone destruction in the joint.

## 2 Results

### 2.1 LACK_156‐173_ Inhibited the Abnormal Activation of RA‐FLSs, Especially the Migration and Invasion

Our previous study demonstrated that LACK_156–173_ attenuated the development of murine autoimmune arthritis by targeting the differentiation, maturation, and functions of DCs. Given that the pathogenesis of RA involves both immune cells and FLSs, it remains unclear whether the LACK protein‐derived peptide can inhibit the activation of RA‐FLSs to suppress disease progression. To investigate the function and specificity of LACK peptides in RA‐FLSs, we evaluated the inhibitory effects of LACK_16‐35_ and LACK_156‐173_ peptides on the production of pro‐inflammatory cytokines by primary human RA‐FLSs. IL‐6 and IL‐8 play critical roles in inflammation and immune responses, while MMP‐1 and VEGFA promote FLS invasion and pannus formation. Compared to PBS and LACK_16‐35_, LACK_156‐173_ significantly reduced the expression of IL‐6, IL‐8, MMP‐1, and VEGFA in TNF‐α‐activated RA‐FLSs, independently of apoptosis (Figure S1A,B, Supporting Information). Thus, our study focused on investigating the therapeutic role of LACK_156‐173_ in targeting RA‐FLSs.

**Figure 1:**
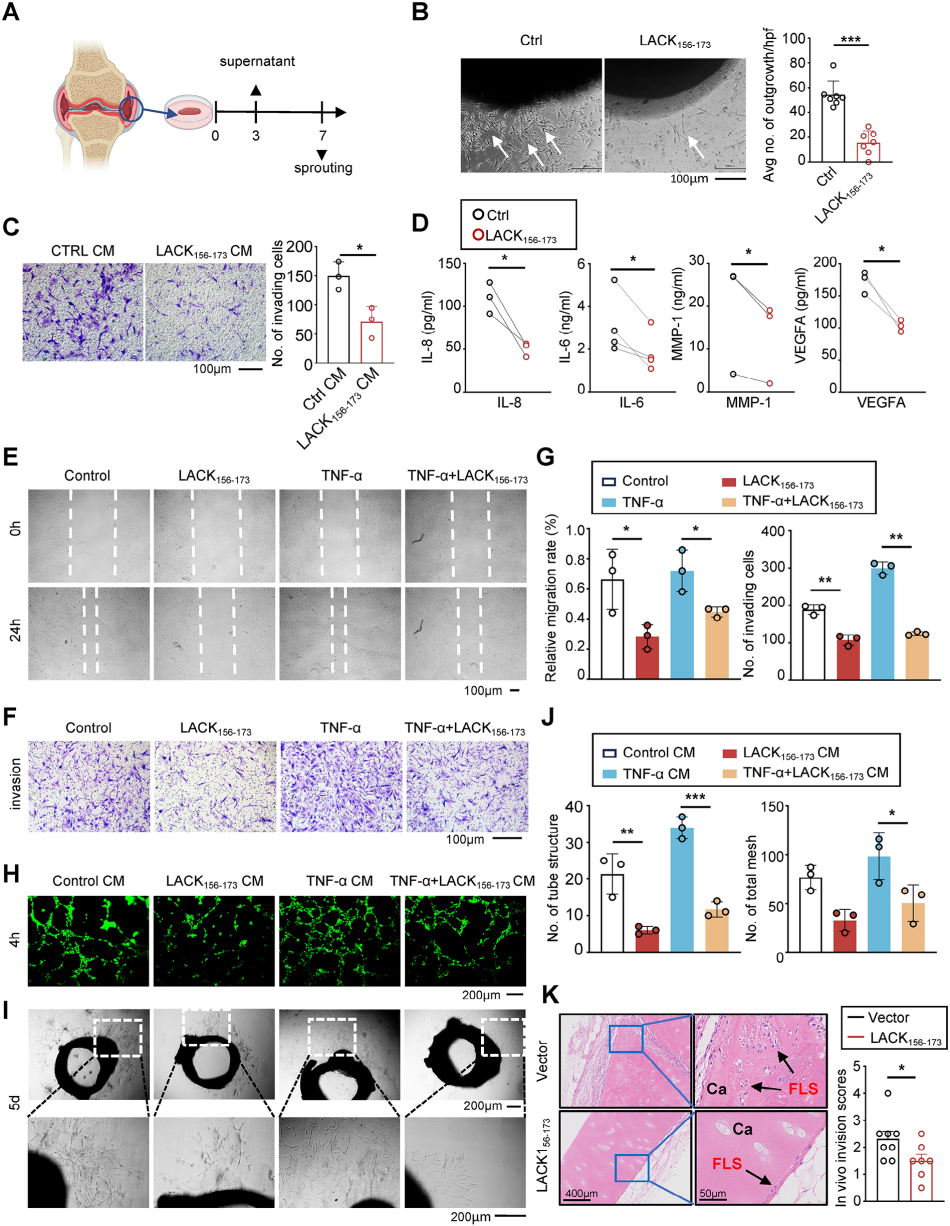
LACK_156‐173_ inhibited the abnormal activation of RA‐FLSs, especially migration and invasion. A) Schematic overview of synovial explants culture. Supernatant was collected after 3 days of cultivation, and RA‐FLS outgrowths were examined on the 7th day. B) Microscopic (left) and statistical analysis (right) of primary RA‐FLS outgrowths from RA synovial explants cultured in Matrigel, following culture with PBS or LACK_156‐173_ for 1 week. The scale bar represents 100 µm. The white arrow indicates RA‐FLS outgrowths (*n* = 7). C) Microscope (left) and statistical analysis (right) of primary RA‐FLSs invasion in response to 50% explant conditioned medium from PBS‐ or LACK_156‐173‐_treated synovial explants (*n* = 3). D) Quantification of the concentration of IL‐8, IL‐6, VEGFA, and MMP‐1 in supernatant from PBS‐ or LACK_156‐173‐_treated synovial explants culture by ELISA (*n* = 3‐4). E‐G) The wound‐healing assay (E) and statistical analysis (G, left) of migration of primary RA‐FLSs 24 h post‐PBS or LACK_156‐173_ treatment. The Transwell assay (F) and statistical analysis (G, right) of the invasion of primary RA‐FLSs 48 h post‐PBS or LACK_156‐173_ treatment. The scale bar represents 100 µm. H–J) The impact of different CMs on angiogenesis was evaluated using tube formation (H) and aortic ring sprouting assays (I). The scale bar represents 200 µm. The total number of tube structures (J, left) and meshes (J, right). K) Histological (left) and statistical (right) analysis of LACK_156‐173′_s effect on primary RA‐FLS invasion into human cartilage implants. Arrows indicate RA‐FLS invasion into cartilage (Ca). Original magnification (left), scale bar represents 400 µm; enlarged magnification (right), scale bar represents 50 µm. Data are mean ± SEM, *n* = 7–8. *p < 0.05, **p < 0.01, and ***p < 0.001 versus vector control, by a two‐tailed unpaired *t*‐test (B,C,K) or paired t‐test (D) or and one‐way ANOVA test (G,J).

To evaluate the therapeutic effect of LACK_156‐173_ on FLS in the preserved tissue microenvironment, an *ex vivo* RA synovial tissue (ST) explant model, which closely reflects the in vivo environment was established (**Figure** **1**A). Treatment with methotrexate (MTX), a clinically established medication for RA, served as the positive control to compare and evaluate the clinical relevance of LACK_156–173_ in the treatment of RA. We observed that the cells within the explant maintained high viability, and the structural integrity of the tissue was preserved after 7 days of in vitro culture (Figure S1C–F, Supporting Information). Consistent with the previous report, LACK_156‐173_ slightly reduced the proportion of CD4^+^CD45RO^+^ and CD8^+^CD45RO^+^ cells by 3.3% and 8.3%, respectively; however, the proportions of other immune cells were not affected (Figure S1G, Supporting Information). Notably, we found that LACK_156‐173_ significantly reduced RA‐ST explant sprouting by 71.48%, which is comparable to MTX (64.3%) (Figure 1B, Figure S1H, Supporting Information), indicating its ability to inhibit RA synovial tissue invasion. The culture supernatant from synovial explants was used as a conditioned medium (CM) for cultivating RA‐FLSs. Compared to the control group, the invasion of RA‐FLSs cultured with LACK_156‐173_‐treated CM was attenuated (Figure 1C). To identify which soluble factors in LACK_156‐173_‐treated CM contribute to the suppression RA‐FLS invasion, we quantified the concentrations of multiple pro‐inflammatory cytokines involved in RA pathogenesis. We found that LACK_156–173_ significantly reduced the spontaneous release of IL‐6, IL‐8, VEGFA, and MMP‐1 by two‐ to threefold compared to the control group (Figure 1D).

Fibroblasts play pivotal roles in maintaining homeostatic tissue function. However, synovial fibroblasts contribute to joint damage and drive angiogenesis in RA. To confirm whether LACK_156‐173_ can directly suppress the invasion and migration of RA‐FLSs, we treated primary RA‐FLSs with either PBS or LACK_156‐173_, and then examined their capacity for invasion by wound healing assay and migration by transwell assay. We found that LACK_156‐173_ can markedly inhibit the migration and invasion of primary RA‐FLSs (Figure 1E–G). The RA synovium pannus formation is composed mainly of fibroblasts, angiogenesis, and immune cells. Increased VEGFA secretion by RA‐FLSs is a key factor in promoting angiogenesis. In RA synovial tissue, upregulated ROS promote pannus formation by inducing hypoxia‐inducible factor mediated VEGF upregulation.^[^ ^4b^ ^]^ We found that LACK_156‐173_ inhibited ROS and VEGFA production by RA‐FLSs (Figure 1D and Figure S1I, Supporting Information). To investigate the effect of RA‐FLSs on angiogenesis, CM from RA‐FLSs was used to induce tube formation in HUVECs and arterial ring sprouting. The number of tube formations and arterial ring sprouting induced by CM from LACK_156‐173_‐stimulated RA‐FLSs was significantly reduced (Figure 1H–J). To assess the in vivo impact of LACK_156‐173_ on RA‐FLS invasion into cartilage, we employed the severe combined immune deficiency (SCID) mouse coimplantation model, in which primary RA‐FLSs with cartilage were co‐implanted into the left and right flanks of SCID mice, and then LACK_156‐173_ was injected locally. We found that LACK_156‐173_ significantly decreased primary RA‐FLS invasion into cartilage compared to PBS‐treated cells (Figure 1K). Taken together, these results indicate that LACK_156‐173_ can directly target RA‐FLSs to attenuate the disease progression of RA by suppressing their invasion and migration capabilities.

### 2.2 LACK_156‐173_ was Internalized into RA‐FLSs via Surface Receptor‐Mediated Endocytosis

**Figure 2:**
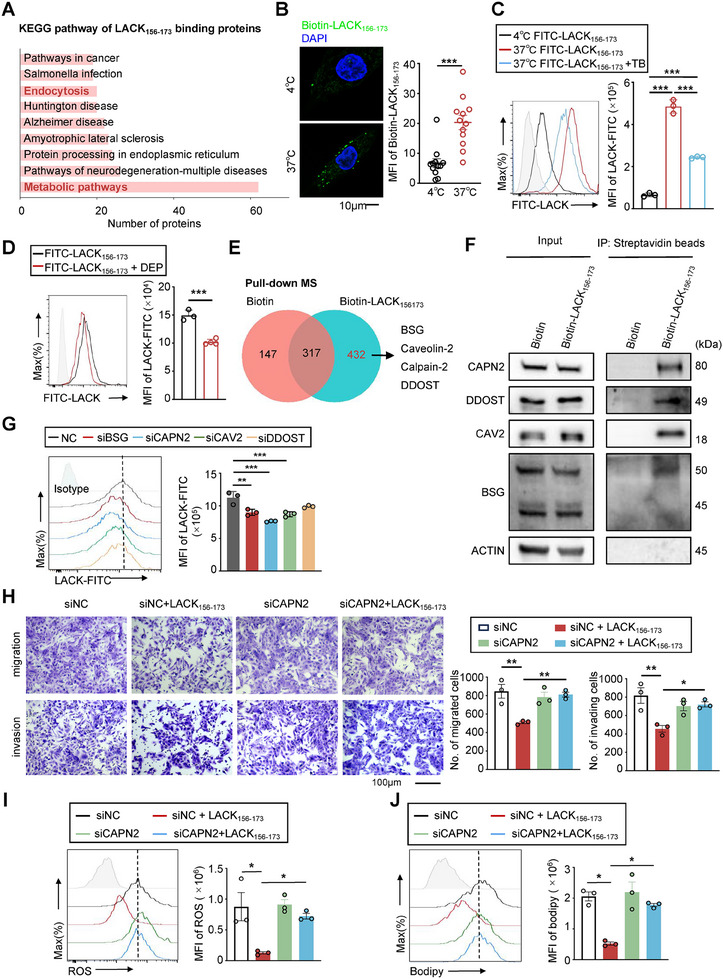
LACK_156‐173_ was internalized into RA‐FLSs via surface receptor‐mediated endocytosis. A) KEGG pathway enrichment analysis of LACK_156‐173_‐interacted proteins identified by mass spectrometry. B) Confocal microscopy (left) and statistical analysis (right) of internalized biotin‐LACK_156‐173_ in primary human RA‐FLS (green). Nuclei were labeled with DAPI (blue). Scale bar = 10 µm. C) Flow cytometry assay (left) and statistical analysis (right) of internalized FITC‐LACK_156‐173_ in MH7A at 4 °C or 37 °C with or without TBs. TB, trypan‐blue. D) Flow cytometry assay (left) and statistical analysis (right) of FITC‐LACK_156‐173_ in MH7A pre‐treated with a cocktail of endocytic inhibitors (DEP, incuding Dynasore, EIPA, and PZB). E) Venn diagram of proteins binding to LACK_156‐173_ in primary human RA‐FLSs identified by mass spectrometry and LACK_156‐173_‐interacted plasma membrane proteins with endocytic properties. F) CO‐IP detection of the interaction of LACK_156‐173_ with CAPN2, BSG, DDOST, and CAV2 in MH7A cells. G) Flow cytometry assay (left) and statistical analysis (right) of FITC‐LACK_156‐173_ in MH7A cells with BSG, CAPN2, CAV2, and DDOST knocked down. H) Transwell assay (left) and statistical analysis (right) of migration and invasion of MH7A with Calpain‐2 knockdown, treated with PBS or LACK_156‐173_ for 48 h. Scale bar represents 100 µm. I,J) Flow cytometry assay (left) and statistical analysis (right) of ROS determined by DCFH‐DA staining (I) and lipogenesis determined by BODIPY 493/503 staining (J) in MH7A cells with Calpain‐2 knocked down, treated with LACK_156‐173_. *p < 0.05, **p < 0.01, and ***p < 0.001 versus vector control, by two‐tailed unpaired *t*‐test (B, D) or one‐way ANOVA test (C,G–J).

The cell membrane acts as a protective barrier and facilitates the transport of small molecules from the extracellular environment through channels or via surface receptor‐mediated endocytosis. To investigate how LACK_156‐173_ is transported into cells, we employed a peptide‐protein pull‐down approach combined with mass spectrometry analysis (Figure S2A, Supporting Information). Mass spectrometry analysis was used to identify the specific proteins interacting with LACK_156‐173_. Enrichment analysis revealed a significant association between LACK_156‐173_ and proteins involved in metabolic and endocytic pathways (**Figure** **2**A). These findings suggest that LACK_156‐173_ can be internalized via endocytosis and potentially influence the metabolism of RA‐FLSs.

To investigate whether RA‐FLSs internalize LACK_156‐173_ through receptor‐mediated endocytosis, we employed various experimental approaches. Initially, we utilized biotinylated LACK_156‐173_ and iFluor488‐labeled streptavidin to examine the subcellular localization and quantification of internalized LACK_156‐173_ through confocal microscopy. We found that RA‐FLSs efficiently take up biotin‐LACK_156‐173_ via endocytosis, with the peptide translocating into the cytosol under 37 °C incubation conditions. In contrast, inhibiting the endocytic pathway by incubating cells at 4 °C markedly blocked the uptake of biotin‐LACK_156‐173_ (Figure 2B). To further validate the interaction between LACK_156‐173_ and the surface proteins of RA‐FLSs, we incubated cells with FITC‐labeled LACK_156‐173_ at 37 °C and subsequently treated them with trypan blue (TB), an aqueous quencher that selectively quenches fluorescence signals from surface‐bound fluorophores.^[^ ^19^ ^]^ This assay revealed that a significant portion of FITC‐LACK_156‐173_ molecules associated with RA‐FLSs was located inside the cells, while the rest remained bound to the cell membrane surface (Figure 2C). Treatment with a cocktail of endocytic inhibitors (Dynasore, EIPA, and PZB) further corroborated these findings by partial inhibition of the uptake of LACK_156‐173_ into RA‐FLSs (Figure 2D). These results collectively indicated that RA‐FLSs take up LACK_156‐173_ peptides predominantly through surface receptor‐mediated endocytosis.

To identify the specific proteins interacting with LACK_156‐173_ and associated with endocytosis, we conducted a detailed analysis of our mass spectrometry data. This analysis revealed that LACK_156‐173_ binds to a total of 432 proteins, with 34 of these being plasma membrane proteins and 213 cytoplasmic proteins (Figure S2B, Supporting Information). From the identified plasma membrane proteins, we focused on those likely involved in endocytosis, leading us to pinpoint four candidates: Basigin (BSG), Caveolin‐2 (CAV2), Calpain‐2 (CAPN2), and Dolichyl‐Diphosphooligosaccharide–Protein Glycosyltransferase Non‐Catalytic Subunit (DDOST) (Figure 2E; Figure S2C,D, Supporting Information). The peptide‐protein co‐immunoprecipitation (Co‐IP) results further confirmed that LACK_156‐173_ interacted with CAPN2, BSG, CAV2, and DDOST in the RA‐FLS cell line MH7A (Figure 2F). Additionally, molecular docking analysis was employed to predict the binding sites between LACK_156‐173_ and its validated binding partners (Figure S3A, Supporting Information). Mutations were introduced at the predicted binding sites on LACK_156‐173_. The LACK_156‐173_ peptides with mutations at the binding sites for BSG, CAPN2, CAV2, and DDOST were designated as LACK‐Mut‐BSG, LACK‐Mut‐CAPN2, LACK‐Mut‐CAV2, and LACK‐Mut‐DDOST, respectively (Figure S3B, Supporting Information). Peptide‐protein pull‐down assays showed that these mutated peptides failed to interact with their corresponding proteins (Figure S3C–F, Supporting Information).

Since ligand binding can induce changes in the thermal stability of target proteins, we performed a cellular thermal shift assay (CETSA) to evaluate the binding intensity between LACK_156‐173_ and BSG, CAPN2, CAV2, and DDOST (Figure S4A, Supporting Information). The results showed that LACK_156‐173_ significantly enhanced the accumulation of CAPN2, DDOST, and BSG within the temperature ranges of 47 °C to 67 °C and 47 °C to 57 °C compared to the DMSO control, while no increase in the accumulation of CAV2 was observed (Figure S4B,C, Supporting Information). Interestingly, the accumulation of CAPN2 was even evident at 72 °C, suggesting CAPN2 could stably interact with LACK_156‐173_ for internalization. Consistently, knockdown of CAPN2 through siRNA resulted in the most significant reduction of LACK_156‐173_ internalization, while silencing DDOST had a minimal effect (Figure 2G). Moreover, when CAPN2 expression was reduced, the inhibitory effects of LACK_156‐173_ on migration and invasion of RA‐FLSs were restored (Figure 2H). Taken together, these findings indicate that RA‐FLSs primarily internalize LACK_156‐173_ through endocytosis mediated by BSG, CAV2, and particularly CAPN2.

### 2.3 LACK_156‐173_ Alleviates the Migration and Invasion of RA‐FLSs by Inhibiting Fatty Acid Synthesis Metabolism

**Figure 3:**
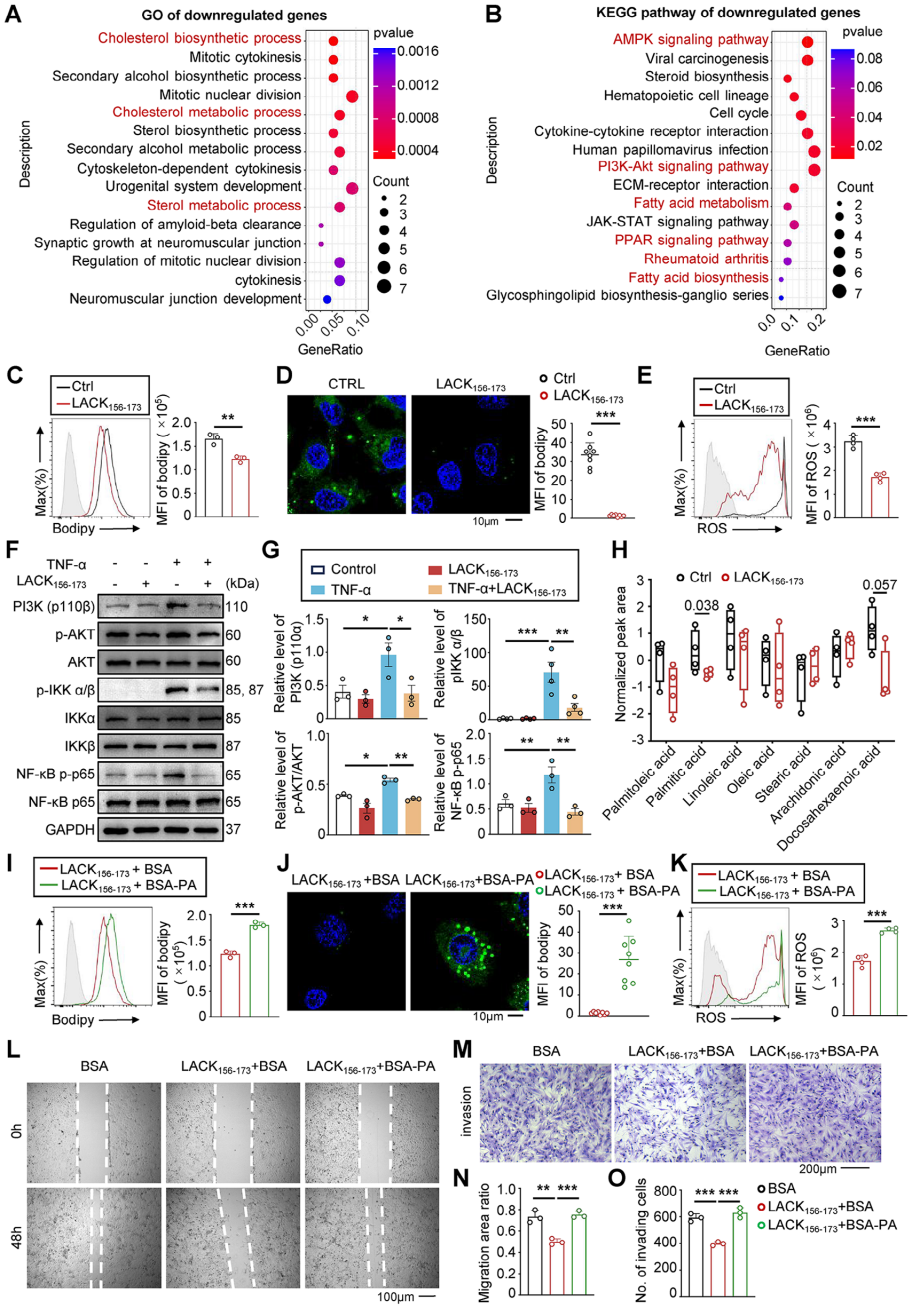
LACK_156‐173_ alleviates the migration and invasion of RA‐FLSs by inhibiting fatty acid synthesis metabolism. A) GO enrichment analysis of DEGs downregulated in LACK_156‐173_ group. Absolute value of fold change ≥ 0.5, *p* < 0.05 and FDR < 0.05, *n* = 3. B) Kyoto Encyclopedia of Genes and Genomes (KEGG) pathway enrichment analysis of DEGs downregulated in LACK_156‐173_ group. Absolute value of fold change ≥ 0.5, *p* < 0.05, and FDR < 0.05 (*n* = 3). C) The flow cytometry assay (left) and statistical analysis (right) of lipogenesis in MH7A cells determined by BODIPY 493/503 staining at 48 h post‐PBS or LACK_156‐173_ treatment. D) Confocal microscopy (left) and statistical analysis (right) of intracellular lipid droplets stained with BODIPY 493/503 staining (green) at 48 h post‐PBS or LACK_156‐173_ treatment. Nuclei were labeled with DAPI (blue). Scale bar = 10 µm. E) The flow cytometry assay (left) and statistical analysis (right) of ROS determined by DCFH‐DA staining. F,G) Western blot assay (F) and statistical analysis (G) of PI3K (p110β), AKT, IKKα, IKKβ, NF‐κB p65 and phosphorylation of AKT, IKKα/β, NF‐κB p65 expression in primary human RA‐FLSs treated with PBS or LACK_156‐173_ (*n* = 3). H) Normalized peak area of the selected metabolites related to lipid biosynthesis normalized to CTRL (MH7A, *n* = 4). I) The flow cytometry assay (left) and statistical analysis (right) of lipogenesis determined by BODIPY 493/503 staining in LACK_156‐173_‐treated MH7A cells with or without palmitate. J) Confocal microscopy (left) and statistical analysis (right) of intracellular lipid droplets stained with BODIPY 493/503 staining (green) in LACK_156‐173_‐treated‐MH7A cells with or without palmitate. Nuclei were labeled with DAPI (blue). Scale bar = 10 µm. K) The flow cytometry assay (left) and statistical analysis (right) of ROS determined by DCFH‐DA staining. L–O) The wound‐healing assay (L) and statistical analysis (N) of migration of MH7A cells 24 h post‐PBS or LACK_156‐173_ treatment with or without palmitate. The scale bar represents 100 µm. The Transwell assay (M) and statistical analysis (O) of invasion of MH7A cells 48 h post‐LACK_156‐173_ treatment with or without palmitate. The scale bar represents 200 µm. *p < 0.05, **p < 0.01, and ***p < 0.001 versus vector control, by two‐tailed unpaired *t*‐test (C–E,H,I–K) or one‐way ANOVA test (G,N,O).

The metabolic dysfunction of RA‐FLSs leads to excessive proliferation, increased invasiveness, and persistent exacerbation of joint inflammation.^[^ ^12^, ^20^ ^]^ To elucidate the underlying mechanism of how LACK_156‐173_ inhibits the activation of RA‐FLSs, we conducted integrated RNA‐seq and metabolomics analyses on PBS or LACK_156‐173_ treated pre‐activate RA‐FLSs. Through GO enrichment analysis, we identified that genes downregulated by LACK_156‐173_ treatment were primarily involved in metabolic pathways, particularly those related to cholesterol biosynthesis, cholesterol metabolism, and sterol metabolic processes (**Figure** **3**A). KEGG pathway analysis highlighted that LACK_156‐173_ treatment attenuated signaling pathways crucial for lipid metabolism such as AMPK, PI3K/AKT, and fatty acid synthesis pathways. Furthermore, it downregulated pathways associated with rheumatoid arthritis pathogenesis (Figure 3B). Our mass spectrometry data indicated that cytoplasmic proteins interacting with LACK_156‐173_ were predominantly associated with metabolic pathways (Figure 2A). To identify which detailed metabolites are affected by LACK_156‐173_ in RA‐FLSs, non‐targeted metabolomics analysis was conducted. To eliminate the potential impact of RA‐FLS heterogeneity from different patients, we utilized the MH7A cell line to assess the effect of LACK_156‐173_ on metabolite levels. Non‐targeted metabolomics analysis identified 223 metabolites, revealing that LACK_156‐173_ treatment significantly altered pathways related to De Novo Triacylglycerol Biosynthesis, a critical pathway in lipid synthesis metabolism (Figure S5A, Supporting Information). To quantify the intracellular neutral lipid content, LACK_156‐173_ treated RA‐FLSs were stained with Bodipy 493/503 and examined by flow cytometry and confocal microscopy. Notably, RA‐FLSs cultured with LACK_156‐173_ displayed significantly less lipid content compared to PBS control based on the MFI of incorporated Bodipy 493/503 (Figure 3C,D). Furthermore, knockdown of CAPN2 restored the inhibitory effects of LACK_156‐173_ on fatty acid content and ROS activity in MH7A cells (Figure 2I,J). These results suggest that LACK_156‐173_ inhibits fatty acid synthesis metabolism.

Mitochondria are organelles that require a coordinated supply of newly synthesized lipids and proteins to function, but excessive accumulation of lipids leads to impaired mitochondrial function and increased ROS, thus, attenuated fatty acid synthesis metabolism could repair damaged mitochondria and downregulate ROS in RA‐FLSs. Previous studies showed that ROS and mitochondrial damage influence FLS proliferation, invasion, and inflammatory factor production, as well as impact FLS survival through processes like apoptosis and autophagy.^[^ ^21^ ^]^ Treatment of RA‐FLSs with LACK_156‐173_ resulted in reduced ROS production (Figure 3E). ROS can activate several pro‐inflammatory signaling pathways, such as AKT and NF‐κB.^[^ ^22^ ^]^ Stimulation of RA‐FLSs with TNF‐α can rapidly increase the expression of PI3K (p110β) and the phosphorylation of AKT, NF‐κB, and IKK α/β, whereas treatment with LACK_156‐173_ alleviates the stimulatory effect of TNF‐α on these pro‐inflammatory signaling pathways (Figure 3F,G). To identify the synthesis of which group of fatty acids was suppressed by LACK_156‐173_, cellular metabolites were analyzed using LC‐MS. The results revealed a significant decrease in a range of free fatty acids (FFAs) in LACK_156‐173_‐treated RA‐FLSs, with palmitic acid showing the most pronounced reduction (Figure 3H). To confirm whether reduced fatty acid metabolism in LACK_156‐173_‐treated FLSs inhibits their activation and migration, exogenous palmitic acid was supplemented to the culture medium of MH7A cells, and its effect on MH7A cells was examined. Addition of palmitic acid reversed the attenuating effects of LACK_156‐173_ on fatty acid content, ROS activity, cell migration, and invasion (Figure 3I–O). Thus, these findings indicate that LACK_156‐173_ could potentially suppress fatty acid synthesis metabolism, restore lipid metabolism balance, and reduce the activation of the AKT/NF‐κB pathway induced by ROS, ultimately attenuating the aggressive phenotype of RA‐FLSs.

### 2.4 LACK_156‐173_ Regulates the Fatty Acid Synthesis by Inhibiting the Enzyme Activity of FASN

**Figure 4:**
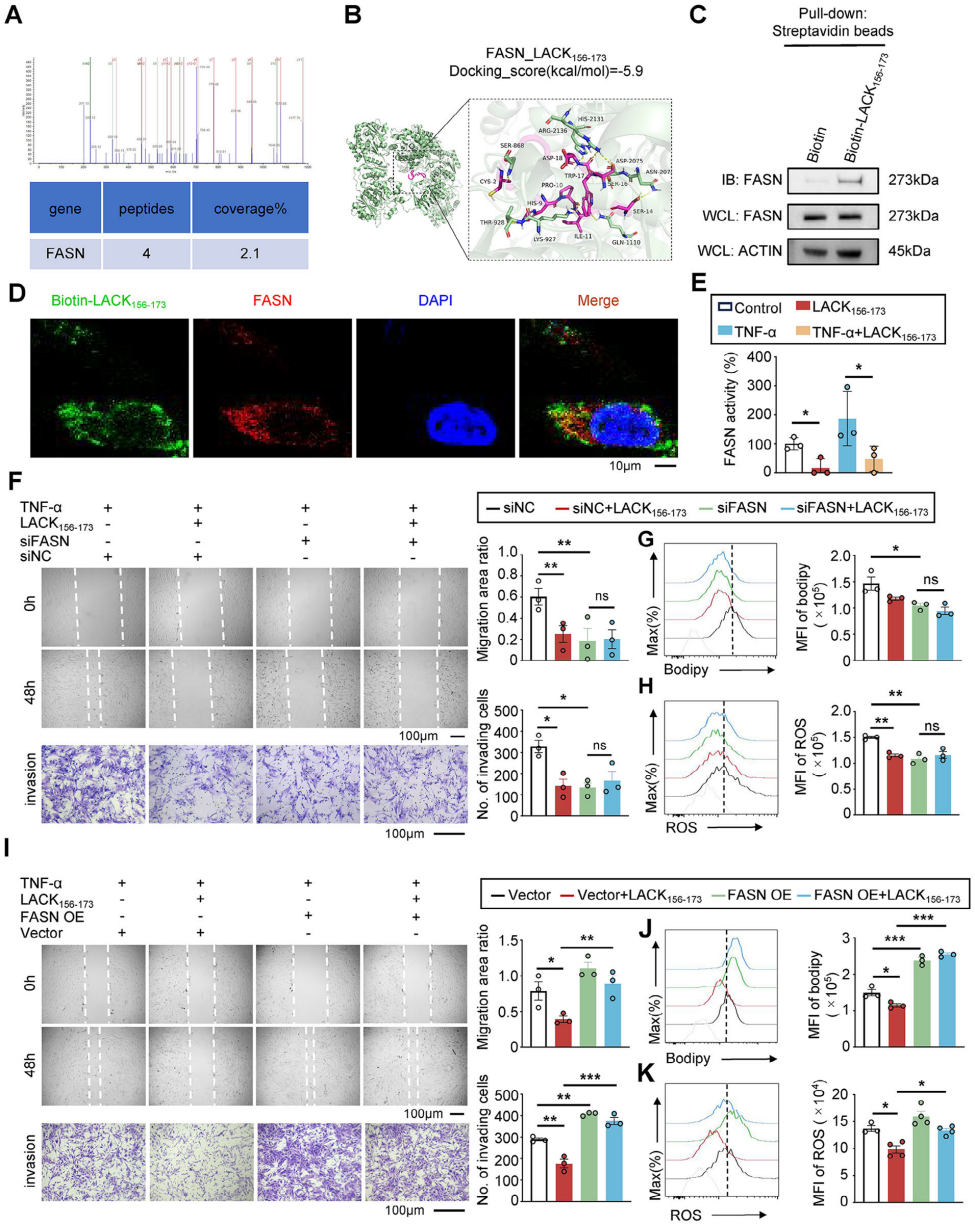
LACK_156‐173_ regulates fatty acid synthesis by inhibiting the enzyme activity of FASN. A) Secondary mass spectra of FASN in mass spectrometry datasets from LACK_156‐173_ pull‐down assay. B) Docking molecular models of LACK_156‐173_ and FASN. C) Immunoblot analysis of FASN in peptide‐protein pulldown assay with biotinylated LACK_156‐173_. D) Confocal microscopy of FASN (red) and biotin‐LACK_156‐173_ (green) colocalization in RA‐FLSs. Nuclei are stained with DAPI (blue). Scale bar represents 10 µm. E) The UV spectrophotometer assay at 340 nm wavelength reflects the enzyme activity of FASN via NADPH. F) The wound‐healing assay (top panel) of migration and Transwell assay (bottom panel) of invasion of RA‐FLSs with or without knockdown FASN 48 h post‐PBS or LACK_156‐173_. Scale bar represents 100 µm. G,H) The flow cytometry assay (left) and statistical analysis (right) of lipogenesis determined by BODIPY 493/503 staining (G), and ROS determined by DCFH‐DA staining in RA‐FLSs (H), with or without knockdown FASN 48 h after PBS or LACK_156‐173_ treatment. I) The wound‐healing assay (top panel) of migration and Transwell assay (bottom panel) of invasion of RA‐FLSs with or without FASN overexpression 48 h post‐PBS or LACK_156‐173_. Scale bar represents 100 µm. J,K) The flow cytometry assay (left) and statistical analysis (right) of lipogenesis determined by BODIPY 493/503 staining (J) and ROS determined by DCFH‐DA staining (K) in MH7A cells with or without FASN overexpression 48 h post‐PBS or LACK_156‐173_ treatment. *p < 0.05, **p < 0.01, and ***p < 0.001 vs vector control. The *p‐*values were determined by a one‐way ANOVA test.

To investigate how internalized LACK_156‐173_ regulates fatty acid synthesis metabolism in RA‐FLSs, we initially examined whether LACK_156‐173_ can directly bind to fatty acid synthesis enzymes using our mass spectrometry datasets from LACK_156‐173_ pull‐down assays. FASN, the key enzyme that catalyzes the synthesis of palmitic acid, was among the cytoplasmic proteins pulled down by LACK_156‐173_, suggesting a direct interaction between internalized LACK_156‐173_ and FASN (**Figure** **4**A). The molecular docking also predicted the interaction between LACK_156‐173_ and FASN (Figure 4B). The direct interaction between LACK_156‐173_ and FASN was confirmed by CETSA and peptide‐protein pulldown (Figure 4C; Figure S4B,C, Supporting Information). The physiological interaction between LACK_156‐173_ and FASN in live MH7A cells was further validated through peptide‐protein Co‐IP and confocal microscopy (Figure 4D; Figure S5B, Supporting Information). Lastly, mutations at key amino acid sites on LACK_156‐173_ severely blocked the interaction with FASN compared to the WT‐LACK_156‐173_ peptide (Figure S5C,D, Supporting Information).

The interaction between LACK_156‐173_ and FASN could potentially lead to protein degradation, downregulation of gene transcription, blockade of enzyme activity, and may even alter the expression of other key enzymes in the fatty acid synthesis pathway. De novo fatty acid synthesis involves four key catalytic enzymes: ATP citrate lyase (ACLY), Acetyl‐CoA carboxylase (ACC), FASN, and stearoyl‐CoA desaturase 1 (SCD1) (Figure S5E, Supporting Information). These enzymes play a critical role in the synthesis and regulation of fatty acids, particularly in the context of inflammation, which is a major driver of disease progression in RA. To distinguish these possibilities, we assessed the protein expression and gene transcription of the four key catalytic enzymes in the fatty acid synthesis pathway. We observed no significant changes in their protein expression, with only minimal reductions in their gene transcription upon LACK_156‐173_ treatment (Figure S5F,G, Supporting Information). FASN catalyzes Acetyl‐CoA, Malonyl‐CoA, and NADPH to generate long‐chain fatty acid and NADP^+^. NADPH, not NADP^+^, can be detected at 340 nm wavelength by UV spectrophotometer. By monitoring the decrease in absorbance at 340 nm over time, the enzyme activity of FASN was quantified based on the rate of NADPH consumption. We found that treatment with LACK_156‐173_ almost completely abolished the catalytic activity of FASN compared to the PBS control (Figure 4E). We further investigated the inhibitory effects of LACK_156‐173_ on the downstream signaling amplifiers of FASN, such as the AMPK and mTOR signaling pathways. LACK_156‐173_ selectively reduced mTOR phosphorylation, while AMPK activation remained unaffected. Notably, the expression of SREBP1, which can induce the expression of FASN was not altered by LACK_156‐173_ and even showed a slight increase (Figure S5H, Supporting Information). Taken together, these results demonstrate that internalized LACK_156‐173_ can directly bind to FASN and inhibit its enzyme activity.

To assess whether inhibition of FASN enzyme activity by LACK_156‐173_ affects RA‐FLS activation, we knocked down FASN expression using siRNA. We designed three different siRNA oligonucleotide sequences targeting FASN. While all three exhibited varying efficacies in reducing FASN levels, siRNA‐1 displayed the strongest inhibitory effect (Figure S6A,B, Supporting Information). Therefore, we selected FASN siRNA‐1 (siFASN) for functional experiments. Knockdown of FASN resulted in reduced migration and invasion of RA‐FLSs (Figure 4F; Figure S6C,D, Supporting Information), decreased lipid content and ROS production (Figure 4G,H). Interestingly, the addition of LACK_156‐173_ did not further reduce migration and invasion of RA‐FLSs or decrease lipid content and ROS levels, suggesting that FASN knockdown had already significantly inhibited these processes to a minimal level. Conversely, overexpression of FASN completely restored the inhibitory effect of LACK_156‐173_ on the migration and invasion, lipid content, and ROS generation of RA‐FLSs (Figure 4I–K; Figure S6E, Supporting Information). Together, these results indicate that FASN acts as a downstream target of LACK_156‐173_ in inhibiting fatty acid synthesis, migration, and invasion of RA‐FLSs.

### 2.5 RA Patients Exhibited Elevated Expression of FASN in FLSs and Synovial Tissues

**Figure 5:**
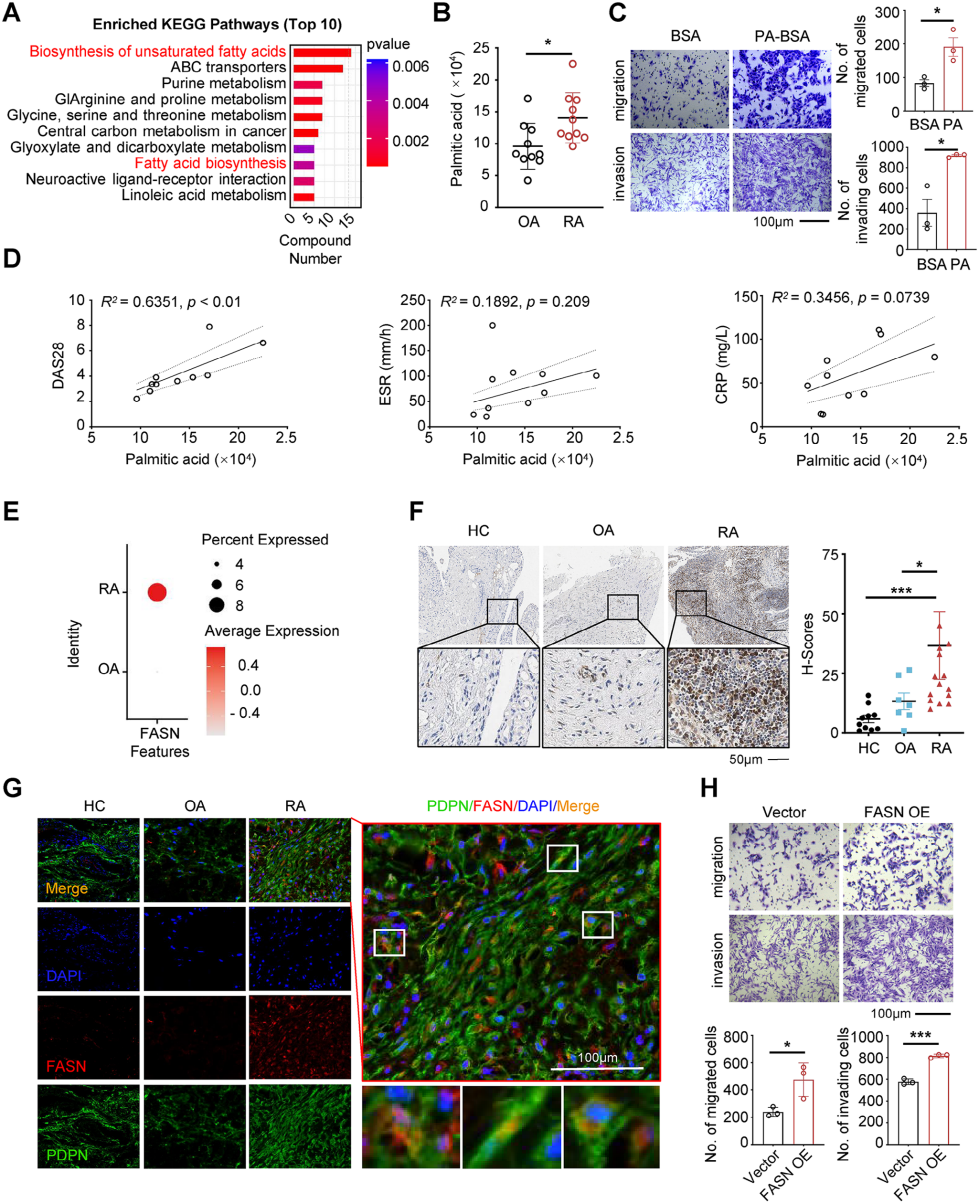
RA patients exhibited elevated expression of FASN in FLSs and synovial tissues. A) KEGG pathway enrichment analysis of different metabolites upregulated in RA synovium versus OA synovium with fold change ≥1.5, *p* < 0.05, and FDR < 0.05 (*n* = 10 biologically independent synovium). B) Statistical analysis of palmitic acid content in synovial tissues of RA and OA. C) Transwell assay of migration (top) and invasion (bottom) of RA‐FLSs treated with BSA or PA for 48 h. Scale bar represents 100 µm. D) The correlation between palmitic acid (PA) and DAS28, erythrocyte sedimentation rate (ESR), and C‐reactive protein (CRP), respectively. E) Bubble diagram shows the expression level of FASN in RA and OA FLSs, respectively. F) Histological (left) and statistical analysis (right) of FASN staining in HC (*n* = 10), OA (*n* = 7) and RA synovium (*n* = 15). The regions in black boxes are shown at higher magnification. Scale bar represents 50 µm. G) Representative multi‐color immunofluorescence images of synovium from RA patients, OA patients and HC, *n* = 3. Scale bar represents 100 µm. H) Transwell assay of migration (top) and invasion (bottom) of RA‐FLSs with FASN overexpression. Scale bar represents 100 µm. *p < 0.05, ***p < 0.001 versus vector control, by a two‐tailed unpaired *t*‐test (B,C,H) or nonparametric Spearman correlation (D) or one‐way ANOVA test (F).

Targeting FASN‐mediated de novo fatty acid synthesis can suppress the hyperactivation of RA‐FLSs; this indicates that dysregulated lipid metabolism in RA‐FLSs may play a pivotal role in the pathogenesis of RA. However, the precise role of fatty acids in the pathogenesis of RA remains unclear. Thus, we investigated whether lipid metabolism is dysregulated in the synovial tissue from RA patients. We conducted metabolomics analysis using samples from individuals with RA (*n* = 10) and osteoarthritis (OA) (*n* = 10) who underwent joint replacement or arthroscopic surgery. Our analysis revealed distinct metabolite profiles in the synovial tissue between RA and OA groups, particularly enriched in lipid metabolism pathways such as biosynthesis of unsaturated fatty acids and fatty acid biosynthesis (absolute fold change ≥ 1.5, *p* < 0.05, and FDR < 0.05) (**Figure** **5**A). Among these metabolites, PA emerged as a key substrate directly catalyzed by FASN for lipid synthesis. Importantly, PA levels were significantly elevated in the synovial tissue of RA patients (Figure 5B). To assess whether dysregulated PA production contributes to the hyperactivation of RA‐FLSs, we cultured RA‐FLSs with exogenous PA and conducted migration and invasion assays. Our results showed that PA treatment significantly increased migration and invasion efficiency by approximately twice compared to the PBS control (Figure 5C). Additionally, there was a positive correlation between PA levels and disease activity scores (DAS28), erythrocyte sedimentation rate (ESR), and C‐reactive protein (CRP) levels in RA patients (Figure 5D).

To assess the clinical significance of FASN in RA, we conducted comprehensive analyses using published datasets of single‐cell sequencing (SDY998, GSE246416, and GSE200815) downloaded from the ImmPort and GEO database.^[^ ^23^ ^]^ We aimed to determine whether FASN gene transcription was elevated in RA‐FLS by comparing its expression in curated and reannotated CELLxGENE single‐cell human cell atlas data. Our analysis revealed a significant upregulation of FASN in FLS from RA patients compared to OA patients and healthy controls (HC) (Figure 5E; Figure S6F, Supporting Information). To validate these findings at the protein level, we performed immunohistochemistry on synovial tissues obtained from RA patients, OA patients, and HC (knee joint synovium from patients with trauma/meniscus injury). Immunohistochemical staining demonstrated higher expression of FASN in RA synovial tissue compared to HC and OA tissues (Figure 5F). Furthermore, to confirm the specific upregulation of FASN in RA‐FLS, we conducted multi‐color immunohistochemistry experiments focusing on podoplanin (PDPN)‐expressing FLS in the synovium from RA patients, OA patients, and HC. Consistently, FASN expression was markedly higher in RA‐FLS compared to OA‐FLS and HC‐FLS (Figure 5G). To explore whether the increased expression of FASN contributes to the hyperactivation of RA‐FLSs, we overexpressed FASN in RA‐FLSs via lentiviral transduction and performed migration and invasion assays. Indeed, overexpression of FASN resulted in enhanced migration and invasion capabilities of RA‐FLSs (Figure 5H). In summary, our data strongly indicate that FASN is significantly upregulated in RA synovial tissue and RA‐FLSs, FASN and its metabolic product phosphatidic acid positively modulate the aggressiveness of RA‐FLSs.

### 2.6 Inhibition of FASN Alleviates Joint Damage in CIA Mice and Inhibits the Invasion of RA‐FLSs

**Figure 6:**
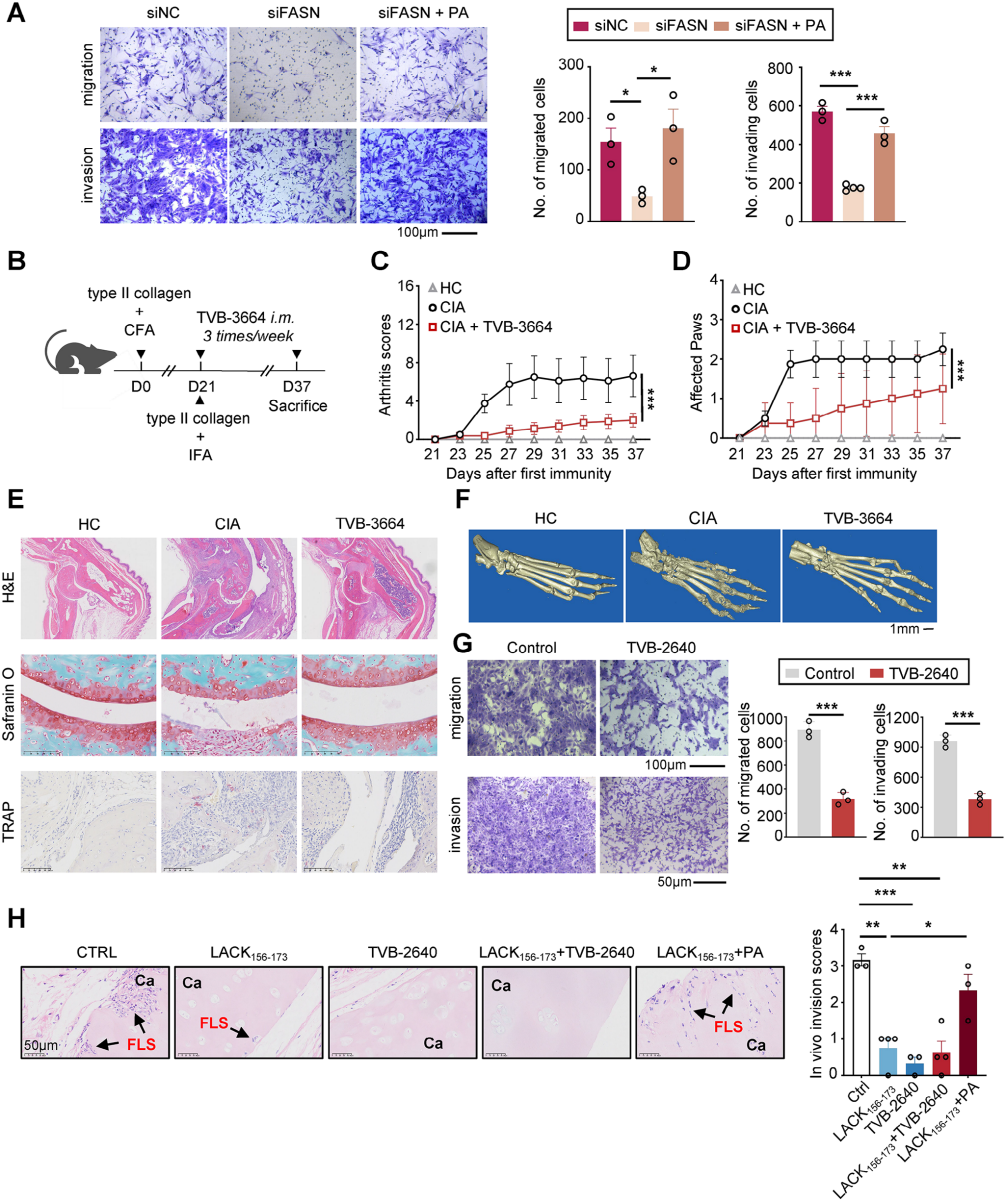
Inhibition of FASN alleviates joint damage in CIA mice and inhibits the invasion of RA‐FLSs. A) Transwell assay of migration (without Matrigel, top) and invasion (with Matrigel, bottom) of MH7A cells with FASN knockdown treated with BSA or PA. Scale bar represents 100 µm. B) Mouse modeling and administration experimental design. After established CIA, the mice were randomized and treated with corn oil or 6mg k^−1^g TVB‐3664 three times/week. C‐D) Statistical analysis of CIA clinical scores and affected paws in CIA mice treated with TVB‐3664. E) Representative histological images of hind paws after staining with hematoxylin and eosin (H&E, scale bar represents 625 µm), Safranin O/Fast green (scale bar represents 100 µm), and TRAP staining (scale bar represents 100 µm). *n* = 8. F) Representative micro‐CT images of ankle joints and hind paws of mice (*n* =  3). Scale bar represents 1 mm. G) Transwell assay of migration (without Matrigel, top) and invasion (with Matrigel, bottom) of MH7A cells treated with TVB‐2640. Scale bar represents 100 µm (up) and 50 µm (down). H) Histological (left) and statistical analysis (right) of the invasion of RA‐FLSs into human cartilage implants. Arrows indicate RA‐FLS invasion into cartilage (Ca). Scale bar represents 50 µm. *n* = 3‐4. *p < 0.05, **p < 0.01, and ***p < 0.001 versus vector control, by one‐way ANOVA test (A, H) or two‐tailed unpaired t‐test (G) or two‐way ANOVA test (C, D).

Although FASN has been extensively studied in tumors and liver diseases, research regarding its role in autoimmune diseases remains unclear. As we have shown, overexpression of FASN and exogenous addition of PA promoted migration and invasion of RA‐FLSs; conversely, knockdown of FASN reduced their migration and invasion. Notably, the addition of PA completely reversed the inhibitory effect of knockdown FASN (**Figure** **6**A). Several inhibitors targeting FASN have been developed, such as C75, Orlistat, TVB‐2640, and TVB‐3664, among others. To investigate the therapeutic potential of targeting FASN in RA, we utilized two commercially available inhibitors, TVB‐2640 and TVB‐3664, in our experiments. TVB‐2640, known for its efficacy in human cells, was primarily used for our in vitro studies.^[^ ^24^ ^]^ TVB‐3664, which demonstrates high potency against mouse FASN,^[^ ^25^ ^]^ was employed in in vivo studies.

To confirm the in vivo therapeutic effect of targeting FASN in RA murine model, we treated collagen‐induced arthritis (CIA) mice with TVB‐3664 (6 mg kg^−1^, administered intramuscularly three times a week starting at booster immunity) (Figure 6B). Notably, treatment with TVB‐3664 led to a substantial decrease in arthritis scores and a reduction in the number of affected joints, the addition of exogenous PA completely reversed the inhibitory effect of TVB‐3664 (Figure 6C,D and Figure S7A,B, Supporting Information). To further evaluate the therapeutic effects and clinical relevance of TVB‐3364 in the treatment of RA, an MTX treatment group was included as a positive control. The therapeutic effect of TVB‐3364 was comparable to that of MTX, as both treatments significantly reduced arthritis scores, from 5.83±3.02 to 1.83±2.61 for TVB‐3364 and 0.71 ± 0.88 for MTX. Additionally, both treatments reduced the median number of affected joints from 2 to 0 (Figure S7A,B, Supporting Information). Histological analysis of joint sections further demonstrated marked improvement in inflammatory infiltration, arthritis‐associated cartilage damage, and bone destruction in TVB‐3664‐treated CIA mice (Figure 6E). Micro‐CT results confirmed significant reduction in bone destruction within the joints of TVB‐3664‐treated mice (Figure 6F). Importantly, the administration of TVB‐3664 neither affects the weight of mice, nor causes any tissue or function damage to kidneys or liver (Figure S7C–E, Supporting Information).

To further extend the clinical relevance of targeting FASN in RA, TVB‐2640 was utilized for human‐related in vitro and in vivo experiments. Treatment with TVB‐2640 reduced the in vitro migration and invasion of human RA‐FLS cell line MH7A (Figure 6G). In the SCID‐cartilage transplant model, TVB‐2640 and LACK_156‐173_ displayed a comparable inhibitory effect on the invasion of human primary RA‐FLSs into cartilage. Combined treatment with LACK_156‐173_ and TVB‐2640 did not show a stronger inhibitory effect. Importantly, supplementation of exogenous PA fully restored the invasion of primary RA‐FLSs into cartilage that was inhibited by LACK_156‐173_ (Figure 6H). This result further supported that LACK_156‐173_ inhibited the invasion of human RA‐FLS through suppression of FASN‐mediated fatty acid metabolism. In conclusion, our findings suggest that targeting FASN holds promise as a novel therapeutic approach for treating RA.

### 2.7 FASN Induces Mitochondrial Fission by Promoting the Phosphorylation of DRP1

**Figure 7:**
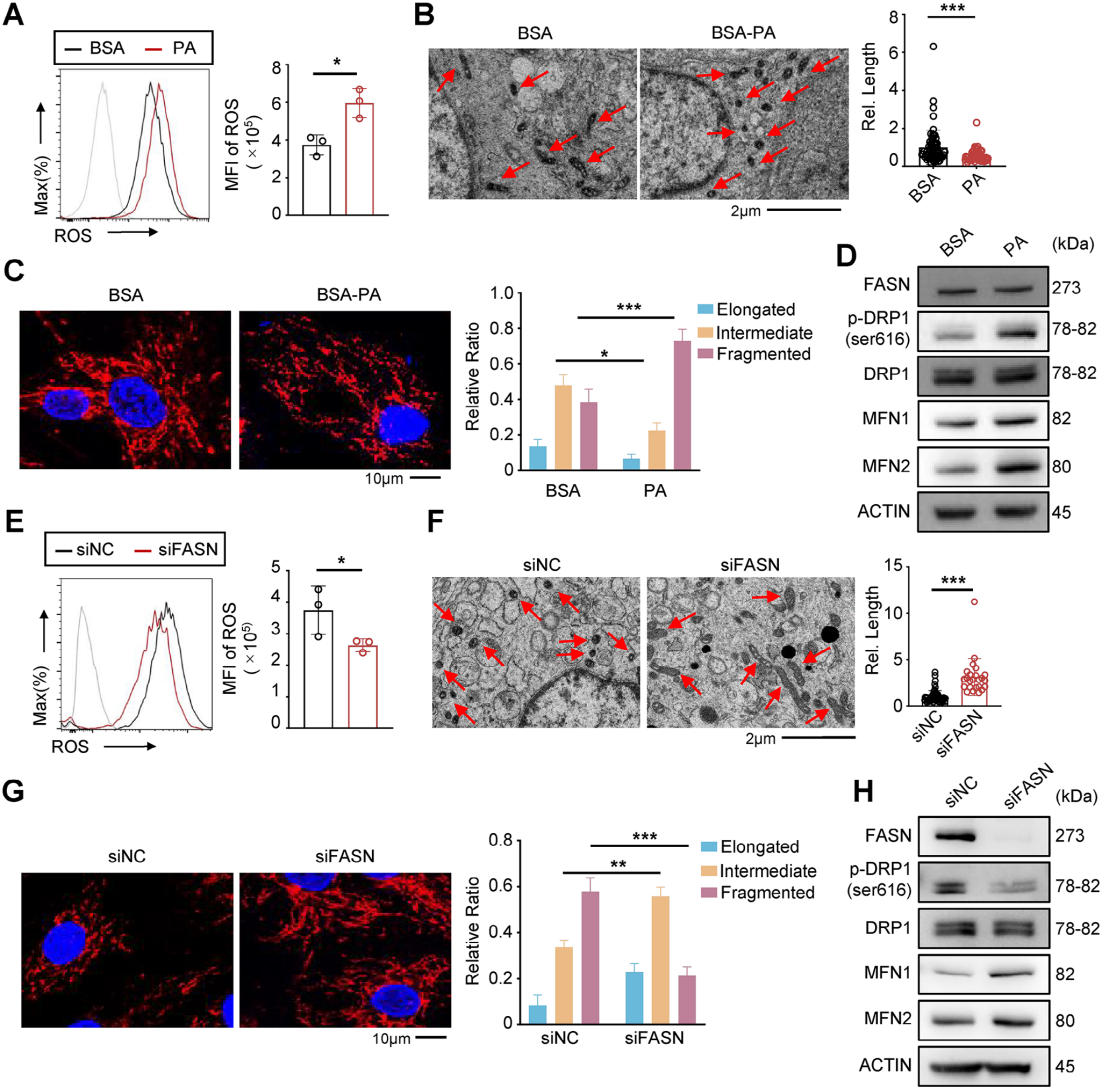
FASN induces mitochondrial fission by promoting the phosphorylation of DRP1. A) The flow cytometry assay (left) and statistical analysis (right) of ROS determined by DCFH‐DA staining in RA‐FLSs after BSA or BSA‐PA treatment. B) Ultrastructure of mitochondria in RA‐FLS cells post BSA or BSA‐PA treatment. Mitochondrial size was measured in three micrographs, with the number of mitochondria being 65. Scale bar represents 2 µm. C) Confocal microscopy (left) and statistical analysis (right) of mitochondria was visualized with anti‐Tom20 antibody (red) in RA‐FLSs with BSA or BSA‐PA treatment. Nuclei are stained with DAPI (blue). Scale bar represents 10 µm. Mitochondrial morphology was analyzed in 300–600 cells across three independent experiments. D) Western blot analysis of FASN, DRP1, MFN1, MFN2, and phosphorylation of DRP1 expression in RA‐FLSs with BSA or BSA‐PA treatment. E) The flow cytometry assay (left) and statistical analysis (right) of ROS determined by DCFH‐DA staining in RA‐FLSs with or without knockdown FASN. F) The ultrastructure of mitochondria in RA‐FLSs with or without knockdown FASN. Mitochondrial length was assessed using three images, with 24–56 mitochondria analyzed. Scale bar represents 2 µm. G) Confocal microscopy (left) and statistical analysis (right) of mitochondria were visualized with anti‐Tom20 antibody (red) in RA‐FLSs with or without knockdown FASN. Nuclei are stained with DAPI (blue). Scale bar represents 10 µm. Mitochondrial morphology was analyzed in 300–600 cells across three experiments. H) Western blot analysis for FASN, DRP1, MFN1, MFN2, and phosphorylation of DRP1 expression in RA‐FLSs with or without knockdown FASN. *p < 0.05, **p < 0.01, and ***p < 0.001 versus vector control, by two‐tailed unpaired t‐test (A, B, E, F) or one‐way ANOVA (C, G).

Normally, fatty acid synthesis mediated by NADK and FASN regulates lipid storage and mitochondrial function.^[^ ^16^ ^]^ But excessive synthesis of fatty acids leads to lipid accumulation, which could drive mitochondrial dynamic equilibrium toward mitochondrial fission.^[^ ^17^ ^]^ Mitochondrial function is fundamental to metabolic homeostasis. Trapped saturated FFAs in the mitochondrial matrix cause ROS production, lipid peroxidation, and mitochondrial dysfunction.^[^ ^26^ ^]^ Our previous study confirmed that excessive mitochondrial fission leading to abnormal activation of RA‐FLS.^[^ ^27^ ^]^ To investigate the impact of FASN and PA on mitochondrial function, we first examined ROS production in RA‐FLSs cultured with exogenous palmitate using flow cytometry. Our results showed a significant increase in ROS production in PA‐treated RA‐FLSs compared with control (**Figure** **7**A). Transmission electron microscopy further provided direct evidence that PA treatment reduced mitochondrial size but increased mitochondrial quantity in RA‐FLSs (Figure 7B). Staining for TOM20, a mitochondrial outer membrane marker, revealed a higher proportion of RA‐FLSs with fragmented mitochondria following PA treatment compared with the control group (Figure 7C). Mitochondrial dynamics are regulated by proteins such as dynamin 1‐like protein (DRP1) and mitochondrial fission protein 1 (FIS1) which regulate fission, and mitofusin 1 (MFN1), MFN2, and Optic Atrophy 1 (OPA1) which regulate fusion.^[^ ^28^ ^]^ Our investigations showed that PA treatment specifically promoted the phosphorylation of DRP1, which indicates enhanced mitochondrial fission activity (Figure 7D). Conversely, knockdown of FASN by siRNA reduced ROS production in RA‐FLSs (Figure 7E). Electron microscopy revealed that FASN knockdown resulted in elongated mitochondria, indicative of enhanced mitochondrial fusion (Figure 7F). Confocal microscopy further supported these findings, showing an increase in RA‐FLSs with elongated mitochondria and a decrease in those with fragmented mitochondria upon FASN knockdown (Figure 7G). Western blot analysis confirmed that FASN knockdown significantly reduced the phosphorylation of DRP1 (Figure 7H). Together, these findings suggest that dysregulated expression of FASN in RA‐FLS facilitates the phosphorylation of DRP1 by catalyzing PA generation, inducing excessive mitochondrial fission and ROS production, eventually leading to the abnormal activation of RA‐FLSs.

## 3 Discussion

RA is characterized by synovial hyperplasia, excessive production of pro‐inflammatory mediators, and aberrant activation of FLS, ultimately leading to joint destruction. However, a subset of patients remains unresponsive to current therapeutic strategies. Increasing evidence suggests that exposure to pathogens, including parasites and protozoa, can influence the immune response in RA.^[^ ^7^, ^29^ ^]^ FLSs are considered a local therapeutic target in RA to mitigate systemic immune suppression associated with current treatments.^[^ ^5^ ^]^ In this study, we identified the parasite‐derived peptide LACK_156‐173_ as a potent modulator of RA‐FLS function, exerting its effects through metabolic reprogramming and inhibition of fatty acid synthesis.

LACK (*Leishmania*‐activated C kinase receptor analog) is a key *Leishmania* antigen involved in immune regulation. Its epitope, LACK_156–173_, is recognized by Vβ4^+^/Vα8^+^ cells, inducing a Th2 response in susceptible BALB/c mice, which impairs parasite control but suggests that the Th2‐polarizing epitope of LACK may have therapeutic potential in autoimmune diseases. Our previous research demonstrated the therapeutic potential of LACK_156‐173_ in collagen antibody‐induced arthritis (CAIA) mice by modulating T cell balance. The pathogenesis of RA also involves non‐immune cells, especially FLSs. The activation of FLSs not only causes tissue damage, but also produces pro‐inflammatory cytokines, including IL‐6 and IL‐8, to recruit and activate innate immune cells. Our study elucidates the novel protective role of LACK_156‐173_, but not LACK_16‐35_, in RA by inhibiting the migration and invasion of RA‐FLSs and suppressing pro‐inflammatory cytokine production. Combined with our previous findings, those results indicate the therapeutic potential of LACK_156‐173_ in RA treatment.

Mechanistically, LACK_156‐173_ enters RA‐FLS via receptor‐mediated endocytosis, primarily involving BSG, CAPN2, CAV2, and DDOST. Although we identified four surface proteins that interact with LACK_156‐173_, CAPN2 exhibited the most stable binding, as shown by CETSA. And knockdown of CAPN2 leads to the strongest inhibition of LACK_156‐173_ uptake, prompting further investigation into its role in suppressing RA‐FLS migration and invasion. CAV2 was thermally unstable, but still contributed to endocytosis at 37 °C, while DDOST showed poor uptake of LACK_156‐173_ with strong binding activity. Knockdown of these receptors did not completely block the internalization of LACK_156‐173_, suggesting the redundant roles of those receptors in the endocytosis of LACK_156‐173_. Interestingly, while the knockdown of CAPN2 partially reduces the uptake of LACK_156‐173_ by RA‐FLSs, it completely abolishes the inhibitory effect of LACK_156‐173_ on RA‐FLS migration and invasion. This result suggests that a high cytosol concentration of LACK_156‐173_ is required to execute its inhibitory function.

Research indicates that free fatty acids (FFA) exert a dose‐dependent effect on RA‐FLSs. FFAs promote the release of key inflammatory mediators, including IL‐6, IL‐8, MCP‐1, and matrix‐degrading enzymes pro‐MMP‐1 and MMP‐3.^[^ ^30^ ^]^ Conversely, polyunsaturated fatty acids like docosahexaenoic acid (DHA) inhibit RA inflammation by reducing TNF‐α and IL‐6 levels in the bloodstream. Metabolomic analysis showed that LACK_156‐173_ restored dysregulated lipid metabolism, including de novo fatty acid synthesis, reducing ROS levels, and attenuating PI3K/mTOR/NF‐κB signaling. The decrease in free fatty acids, particularly palmitic acid, along with rescue experiments, supports metabolic reprogramming as the basis for LACK_156‐173_‐mediated inhibition of RA‐FLS migration and invasion. Additionally, LACK_156‐173_ slightly reduced DHA levels, likely due to compensatory consumption following suppressed fatty acid synthesis. Whether this results from increased DHA utilization or altered catabolism requires further investigation.

Studies have shown that Morin inhibits the differentiation of Th17 cell and the transcription of Fasn in CIA mice, with Fasn overexpression reversing Morin's effects on Th17 differentiation.^[^ ^31^ ^]^ However, the exact role of FASN in FLS and RA remains unclear. This study reveals a direct interaction between LACK_156‐173_ and FASN, a key enzyme in lipid biosynthesis linked to cell growth and signaling (PI3K/mTOR/NF‐κB). The interaction between LACK_156‐173_ and FASN leads to the suppression of FASN‐mediated catalytic enzyme activity, without affecting the protein expression of four key catalytic enzymes (ACLY, ACC, FASN, and SCD1) involved in fatty acid synthesis. LACK_156‐173_ notably suppressed mTOR phosphorylation but showed limited influence on AMPK and SREBP‐1, a key FASN transcription factor.

Based on these findings, we explored the potential of targeting FASN as a therapeutic strategy for RA. Two FASN‐targeted commercially available inhibitors, TVB‐2640 and TVB‐3664, were used in this study. For in vivo murine studies, we opted for TVB‐3664 due to its superior specificity in mice, which alleviates joint damage and synovial hyperplasia, thereby supporting the feasibility of FASN‐targeted therapy for RA. On the other hand, fatty acid oxidation has been implicated in promoting osteoclast formation.^[^ ^32^ ^]^ In RA synovium, osteoclast precursor cells (macrophages) rely on RANKL expressed by FLSs for osteoclastogenesis. Treatment with TVB‐3664 in collagen‐induced arthritis (CIA) mice not only mitigated FLS‐mediated cartilage erosion but also suppressed the destruction of the articular bone surface. This dual effect may stem from TVB‐3664′s inhibition of FLS activity and its potential to attenuate macrophage differentiation into osteoclasts. In the context of the human RA model, treatment with TVB‐2640 or LACK_156‐173_ both significantly attenuated the migration and invasion of primary human RA‐FLS into cartilage. More importantly, this inhibitory effect caused by LACK_156‐173_ was dependent on FASN‐mediated fatty acid metabolism. Those results further indicated the clinical relevance of using LACK_156‐173_ in RA treatment. However, the in vivo stability of LACK_156‐173_ will be determined and optimized in our future study. Given that RA is a systemic inflammatory disease, our future study will also focus on the development of specialized delivery systems, such as nanoparticle‐based system or PEGylation, to improve the stability and therapeutic effect of LACK_156‐173_ in the treatment of RA.

In summary, our study identifies LACK_156‐173_ as a novel modulator of RA‐FLS function, exerting its effects through metabolic reprogramming and direct FASN inhibition. These insights underscore the therapeutic potential of targeting FASN in RA, paving the way for innovative disease management strategies that complement conventional immunosuppressive approaches.

## 4 Conclusion

Dysregulated fatty acid metabolism drives RA‐FLS migration and invasion. LACK_156‐173_ enters cells via receptor‐mediated endocytosis (e.g., CAPN2), directly binds FASN, and inhibits its activity, reducing palmitic acid production. This suppression dampens PI3K/mTOR/NF‐κB signaling, lowers inflammatory cytokine levels, and mitigates RA‐FLS invasiveness, ultimately alleviating arthritis symptoms. These findings underscore the therapeutic potential of LACK_156‐173_ and suggest targeting fatty acid metabolism as a viable strategy for RA treatment.

## 5 Experimental Section

### Patient Samples and Cell Preparation

Human synovial tissue samples were collected from individuals undergoing knee replacement surgery at. Guangdong Provincial People's Hospital and Second Affiliated Hospital of Harbin Medical University. All participants (*n* = 26, aged 28–79; 24 females, 2 males) met the 2010 ACR‐EULAR RA classification criteria.^[^ ^33^ ^]^ Written informed consent was obtained from patients or their legal representatives prior to sample collection. Ethical approval for this research was obtained from both Guangdong Provincial People's Hospital (Approval No.: KY‐Z‐2022‐2520‐02) and Second Affiliated Hospital of Harbin Medical University (Approval No.: KY2020‐205). RA patients’ demographics are provided in Table S1 (Supporting Information). Arthroscopic biopsies of healthy control synovial tissue were collected from a cohort of 10 subjects (8 women and 2 men) aged 39 to 68 years. These individuals underwent arthroscopic surgery for meniscus or cruciate ligament injuries with no history of arthritis. Synovial tissue was minced and digested with 1 mg mL^−1^ collagenase I at 37 °C for 3 h to isolate FLSs. The collagenase digestion was neutralized using DMEM/F12 supplemented with 10% FBS, and the cell suspension was filtered through a 70 µm cell strainer. All cells were cultured in DMEM/F12 containing 10% FBS at 37 °C and 5% CO_2_. In the experiments, primary RA‐FLS from the 3rd to 6th passages were used. Additionally, some experiments involved the human RA‐FLS cell line MH7A, which was derived from primary FLS.

### Ex Vivo RA Synovial Explants

Synovial tissue from RA patients undergoing joint replacement surgery was cut into 5 × 5 mm fragments and cultured in a 96‐well plate pre‐coated with the Matrigel in complete DMEM/F12 medium. This culture model maintains the synovial structure and cell‐cell contacts, providing a closer reflection of the in vivo environment.^[^ ^34^ ^]^ For co‐culture experiments, the explants were cultured in the presence of LACK_156‐173_ (100 µg mL^−1^) or DMSO (1‰) as a control. Each experiment combined three technical replicates (three individual biopsies from one patient) to obtain a biological sample, which was performed across multiple different patients. This is crucial for considering heterogeneity within patient joints and between patients. Following a 72‐h culture period of the explants, the supernatant was collected, centrifuged to remove cell debris, and either used for ELISA analysis or mixed 1:1 with DMEM/F12 medium containing 10% FBS as conditioned medium to culture RA‐FLS for assessing their invasive capacity. Concurrently, the explants themselves were harvested to determine the weight of each viable tissue under examination. For tissue sprouting experiments, the explants were subjected to 7‐day culture to evaluate tissue outgrowth. The activity of synovial explants was assessed by incubating with calcein‐AM for 15 minutes, followed by replacement with fresh culture medium. Images were then captured using a fluorescence microscope.

### Conditioned Medium

Primary RA‐FLS were cultured with LACK_156‐173_, TNF‐α, their combination, or PBS for 48 h, followed by a medium replacement and an additional 48‐hour incubation. The supernatant was collected and centrifuged at 3000 × *g* for 5 min to remove cell debris. The clarified supernatant was either used directly or diluted with fresh medium at a defined ratio to prepare conditioned medium for functional experiments.

### Cytokine Production

Quantification of cytokine levels in the culture supernatants was performed using human Enzyme‐Linked Immunosorbent Assay (ELISA) Kits specific for IL‐8 and VEGFA (obtained from Wuhan ColorfulGene Biological Technology), IL‐6 (obtained from Cusabio), and MMP‐1 (obtained from Boster Biological Technology), following the manufacturer's instructions.

### Transwell Assay

Cell migration and invasion in vitro were measured using a chemotaxis assay with the Transwell chamber method. Transwell chambers with an 8.0 µm pore size were employed (Corning Labware Products). FLS (1 × 10^5^) were resuspended in 200 µL of serum‐free DMEM/F12 and seeded into the upper chamber of the Transwell insert, while DMEM/F12 complete medium containing 10% FBS was added to the lower chamber as a chemoattractant. After incubation at 37 °C in a 5% CO₂ incubator for 24 h, non‐migrated cells on the upper membrane surface were removed using a cotton swab. The inserts were then fixed in 4% paraformaldehyde for 10 min, washed twice with PBS, and stained with 0.1% crystal violet for 15 min. Excess crystal violet was rinsed off with distilled water. Migrated cells on the lower side of the membrane were photographed and counted under a light microscope. The average number of cells in five random fields was used to quantify migration. For the in vitro invasion assay, a similar procedure was followed using inserts precoated with BD Matrigel (BD Biosciences) on the upper membrane surface. The plates were incubated at 37 °C with 5% CO₂ for 48 h.

### Wound Healing Migration

Primary RA‐FLS or MH7A cells were seeded into six‐well plates. When cell confluence reached 80–90%, a 200 µL pipette tip was used to create mechanical scratches. Detached cells were removed by washing the plates three times with PBS, and the remaining cells were cultured in DMEM/F12 or RPMI 1640 medium supplemented with 2% FBS. Images were captured under microscope at 0, 24, and 48 h. Cell migration was assessed by quantifying the scratch area, with the migrated area calculated relative to the initial scratch at 0 h.

### Tube Formation and Aortic ring Sprouting Assays

Pre‐coat the wells of a 24‐well plate with a matrix gel. Digest HUVEC using trypsin, count 20000 cells per well, and seed the cells. After 4 h of cultivation at 37 °C in conditioned medium, stain with calcein‐AM for 15 minutes, replace with culture medium, and capture images under a fluorescence microscope. Analyze the number of tube‐like structures with Image J. The thoracic aorta was isolated from 6‐week‐old Sprague‐Dawley rats, stripped of surrounding connective tissue, and cut into 2 mm‐thick rings. Infiltrate these rings onto a 48‐well plate coated with Matrigel and cultivate them using a mixed medium comprising 50% H‐DMEM (10% FBS) and 50% conditioned medium. After 5 days, capture images and perform a count of the mesh‐like structures.

### RT‐qPCR

Total RNA was extracted using the TRIzol method. Briefly, drug‐treated RA‐FLS were lysed in TRIzol and either stored at ‐80 °C or processed immediately for RNA extraction. After adding chloroform (1/5 volume) and thorough mixing, the mixture was centrifuged at high speed under 4 °C. The supernatant was collected, mixed with an equal volume of isopropanol, and centrifuged to precipitate RNA. The pellet was washed twice with 75% ethanol, air‐dried, and dissolved in RNase‐free water. Reverse transcription was performed using the PrimeScript RT Reagent Kit with gDNA Eraser, followed by RT‐qPCR on a Bio‐Rad CFX96 system. Real‐time PCR primers are listed in Table S2. Gene expression levels were normalized to the endogenous reference gene ACTIN (ΔCt = Ct target – Ct ACTIN) and analyzed using the ΔΔCt method (ΔΔCt = ΔCt sample – ΔCt calibrator). Each experiment was conducted in triplicate.

### Western Blot Analysis

Protein concentrations were measured with the Pierce BCA Protein Assay Kit (Thermo Fisher Scientific), and protein was solubilized in Protein Loading Buffer (TRANS), followed by boiling for 10 minutes. The proteins were then separated by SDS‐PAGE and transferred onto polyvinylidene fluoride (PVDF) membranes (Immobilon). The membranes were probed overnight at 4 °C with primary antibodies, including anti‐PI3K (4249, Cell Signaling Technology), anti‐AKT (4691, Cell Signaling Technology), anti‐phospho‐AKT (4660, Cell Signaling Technology), anti‐IKKα (11 930, Cell Signaling Technology), anti‐IKKβ (8943, Cell Signaling Technology), anti‐phospho‐IKKα/β (2697, Cell Signaling Technology), anti‐NF‐κB p65 (8242, Cell Signaling Technology), anti‐phospho‐NF‐κB p65 (3033, Cell Signaling Technology), anti‐ACC (67 373, Proteintech), anti‐FASN (3180, Cell Signaling Technology), anti‐ACLY (PTM‐20018), anti‐SCD1 (Ab236868), and anti‐ACTIN (4970, Cell Signaling Technology), anti‐AMPK α1 (10929‐2‐AP, proteintech), anti‐phospho‐AMPKα1(2535T, CST), anti‐SREBP1 (14088‐1‐AP, proteintech), anti‐mTOR (66888‐1‐Ig, proteintech), anti‐phospho‐mTOR (67778‐1‐Ig, proteintech), anti‐CAPN2 (66977‐1‐Ig, proteintech), anti‐BSG (66443‐1‐Ig, proteintech), anti‐DDOST (14916‐1‐AP, proteintech), anti‐Caveolin‐2 (PC6310S, Abmart), diluted in Primary Antibody Dilution Buffer (Beyotime). Afterward, the PVDF membranes were incubated with appropriate secondary antibodies at room temperature for 1 hour. Protein bands on the membrane were visualized using Amersham ECL Prime Western Blotting Detection Reagent (GE Healthcare). Each blot represents a minimum of three independent experiments.

### Apoptosis Assays

The assessment of FLS apoptosis involved staining with Annexin V (Fluorescein 5‐isothiocyanate, FITC) and DAPI, following the protocol provided by BD Biosciences. Briefly, 1 × 10⁶ cells were suspended in 100 µL of 1 × binding buffer. Then, 4 µL of FITC Annexin V and 5 µL of DAPI were added to the mixture. Subsequently, the mixture was incubated in darkness at 4 °C for 15 min. Within an hour, the samples were subjected to analysis using flow cytometry.

### Preparation of Single‐Cell Suspensions and Flow Cytometry

Synovial explants treated with either LACK_156‐173_ or vehicle control for 72 h were collected, cut into small pieces with scissors, and dispersed in a 5 mL tube. The tissue was then digested with 1 mg mL^−1^ collagenase I at 37 °C, with shaking at 200 rpm for 30 min. The cell suspension was filtered through a 200‐mesh filter and centrifuged at 450 × *g* for 5 min. The supernatant was aspirated, leaving the cell pellet. For flow cytometry, 1 × 10^6^ cells were selected for leukocyte blocking, antibody staining, and washing. The cell pellet was resuspended in 300 µL of 1 × PBS containing 2% BSA and analyzed using a Cytek 23‐color spectral flow cytometer (Cytek, USA). PBS containing 2% BSA and analyzed using a Cytek 23‐color spectral flow cytometer (Cytek, USA).

### Mice

All mice were kept in specific pathogen‐free conditions, at a stable temperature of 22–26 °C with a relative humidity of 40–65% under a 12‐hour light/dark cycle. The experiments were approved by Guangdong Provincial People's Hospital (Approval No.: KY‐Z‐2022‐2520‐02). All experiments involving mice were conducted in a randomized manner, ensuring unbiased allocation. All data and samples were included in the analyses to ensure comprehensive representation. The figures display individual data points, providing a detailed visual representation. While the data distribution was assumed to be normal, it should be noted that a formal statistical test for normality was not performed.

### Assessment of In Vivo RA‐FLS Invasion into Human Cartilage Implants

To assess the invasive potential of primary RA‐FLS in vivo, cultured cells were trypsinized, resuspended in sterile saline, and adjusted to 100 µL per implant. Human cartilage from non‐arthritic patients undergoing knee surgery for traumatic injuries was cut into 5–8 mm^3^ pieces and snap‐frozen at −80 °C. For implantation, 6‐week‐old SCID mice (GemPharmatech Co., Ltd) were used. On the day of implantation, cartilage fragments were embedded into pre‐incised 80 mm^3^ sponge cubes, which were then soaked with 4 × 10⁵ RA‐FLSs in sterile saline. Two implants containing both FLSs and cartilage were placed subcutaneously into the bilateral flanks of anesthetized mice under sterile conditions. A topical treatment was applied every other day. After 4 weeks, implants were collected from euthanized mice, fixed in 4% paraformaldehyde, and embedded in paraffin. Tissue sections were stained with H&E, and RA‐FLS invasion into cartilage was evaluated using a standardized scoring system: 0 = no or minimal invasion; 1 = shallow invasion (≤2‐cell depth); 2 = moderate invasion (≤5‐cell depth); 3 = deep invasion (>10‐cell depth). Histological assessments were conducted in a blinded manner by two independent investigators.

### Arthritis Induction

Collagen‐induced arthritis (CIA) was established in DBA/1 mice as previously reported.^[^ ^7^ ^]^ Briefly, an emulsion of bovine type II collagen (CII) solution (2 mg mL^−1^ in 0.05 m acetic acid) was prepared by mixing equal volumes of complete Freund's adjuvant (Chondrex, Redmond, WA, USA) and the CII solution (Chondrex, Redmond). On day 0, 0.1 mL of the emulsion was subcutaneously injected at the base of the mouse tail. On day 21, a booster dose (0.1 mL) of CII emulsified in incomplete Freund's adjuvant was administered near the initial injection site. After the booster, mice were assessed every other day until day 37 post‐arthritis induction.

### Histomorphometric Analysis

Joint tissue sections were stained using H&E, Safranin O, and TRAP according to previously described methods.^[^ ^35^ ^]^ Ankle joints were collected from TVB‐3664‐treated and untreated CIA mice, as well as healthy controls, with skin and muscle carefully removed. The samples were fixed in 4% paraformaldehyde for 24 h, followed by decalcification in EDTA decalcification solution (ServiceBio, G1105) at room temperature for 3 weeks. Subsequently, the joints were embedded in paraffin and sectioned into 5 µm slices. H&E staining was performed to assess inflammatory cell infiltration and pannus formation in the synovium. Safranin O‐Fast Green staining was used to evaluate cartilage damage. TRAP staining was conducted using the TRAP Staining Kit (ServiceBio, G1050) according to the manufacturer's instructions to assess bone resorption, followed by hematoxylin counterstaining for nuclear visualization.

### Immunohistochemistry (IHC) and Multiplex Immunohistochemistry (mIHC)

Synovial tissue specimens were fixed in 4% PFA for 24–48 h and then embedded in paraffin. For immunohistochemistry (IHC), 4 µm synovial tissue sections were rehydrated and incubated in 3% H_2_O_2_ for 5 min to block endogenous peroxidase activity. Antigen retrieval was performed by boiling the sections in 0.01 m citrate buffer (pH 6.0). After blocking with 5% goat serum, the sections were incubated overnight at 4 °C with a primary antibody against FASN (CST, 3180, 1:200). The following day, tissue sections were incubated at room temperature for 1 hour with an HRP‐conjugated goat anti‐rabbit secondary antibody. Detection was performed using 3,3′‐diaminobenzidine (DAB, DAKO, CAT#10036913), followed by hematoxylin counterstaining. Imaging was conducted using a pathology slide scanner (Leica, CAT#Aperio CS2).

For multiplex immunohistochemistry (mIHC), sections were incubated overnight with primary antibodies against FASN (CST, 3180, 1:200) or PDPN (Abcam, ab236529, 1:200), followed by a 10‐minute incubation at room temperature with secondary antibodies. Fluorophore labeling was performed using Tyramide Signal Amplification (TSA), with PPD520 for PDPN and PPD620 for FASN (1:100, Panovue, Cat#00044100100), incubated at room temperature for 10 minutes. Subsequently, antigen retrieval was repeated to remove all conjugates except fluorophores for the next staining cycle. Finally, cell nuclei were counterstained with DAPI (Beyotime, China). Slide scanning and imaging were performed using a multiplex tissue imaging and analysis system (PerkinElmer, Cat#Vectra 3).

### Micro‐CT Analysis

Ankle joints and hind paws were separated from mice, fixed in 4% paraformaldehyde for 24 h, and scanned by micro‐CT (90 kV, 88 µA, 10 µm).

### Serum Biochemical Measurements

At the conclusion of the experiment, blood samples were collected from the mice. The samples were centrifuged at 3000 × *g* for 15 min at 4 °C to isolate the serum. Liver toxicity was evaluated by measuring serum levels of aspartate aminotransferase (AST) and alanine aminotransferase (ALT). Similarly, renal toxicity was assessed by quantifying serum levels of creatinine (Crea) and uric acid (UA).

### Metabolite Isolation and LC–MS Analysis

The synovial tissues were collected and snap‐frozen at −80 °C, minced, and lysed. MH7A cells were washed twice with pre‐chilled (4 °C) 1× PBS, followed by lysis using an extraction buffer composed of methanol, acetonitrile, and water at a volume ratio of 7:2:1. Cell disruption was further facilitated using an ultrasonic homogenizer (SCIENTZ, JY92‐IIN, China) for 15 min. The resulting lysate was centrifuged at 15 000 × *g* for 15 min to obtain the metabolite‐containing supernatant. This supernatant was further centrifuged and concentrated using a vacuum centrifuge, yielding a precipitate, which was then reconstituted in an aqueous solution containing 0.1% methanol (50 µL per 1 × 10^6^ cells). For untargeted metabolomic analysis, a Q Exactive Plus mass spectrometer (Thermo, USA) coupled with an HPLC system (Thermo, USA) was used via an electrospray ionization source.

### Detection of FITC‐LACK_156‐173_ Internalization

Cells were incubated with FITC‐labeled LACK_156‐173_ for 4 h, then digested with trypsin and subsequently treated with or without 0.01% trypan blue (TB), an aqueous quencher that selectively reduces fluorescence from surface‐bound fluorophores.^[^ ^19^ ^]^ The flow cytometry assay was performed within 10 min of TB staining.

### Immunofluorescence (IF)

Culture RA‐FLSs with Biotin‐LACK_156‐173_ in a confocal dish (Biosharp, BS‐15‐GJM) for 4 h. Then, fix the cells with 4% PFA for 15 min. Block with a buffer solution (1 × PBS, 5% BSA, 0.3% Triton X‐100) at room temperature for 1 h. Then, incubate the cells with the indicated primary antibody (eg.: FASN 1:200, Abcam), overnight at 4 °C. Following overnight incubation, incubate the cells with Alexa Fluor 594‐conjugated secondary antibodies (CST) and iFluor488‐labeled streptavidin conjugate (AAT Bioquest) in the dark at room temperature for 1 hour, then stain the nuclei with DAPI. Capture the images using a Nikon confocal laser scanning microscope.

### Peptide–Protein Pull‐Down

Perform a Biotin‐LACK_156‐173_ binding assay by incubating it with streptavidin‐coated magnetic beads at 4 °C for 4 h with continuous rotation. Remove unbound peptides by centrifugation at the highest speed. Then, incubate the cell lysate with the magnetic bead‐peptide complex at 4 °C for 12 h with continuous rotation. Specific binding of target protein molecules to the peptide occurs during this step. Non‐specifically bound proteins can be eliminated by performing washing steps. After eluting the peptide‐protein complex using an elution buffer, proceed to separate the complex through SDS‐PAGE electrophoresis. Utilize silver staining to compare the band patterns between the control and experimental groups. To precisely determine the protein types, identify the distinct bands through mass spectrometry (MS). Alternatively, confirm the presence of specific proteins using western blot analysis.

### Co‐Immunoprecipitation (Co‐IP)

2 × 10^6^ MH7A cells were seeded into 25 cm^2^ flasks and allowed to adhere. Biotin or biotinylated Biotin‐LACK_156‐173_ peptide was added to a final concentration of 0.5 mm, and the cells were incubated at 4 °C for 3 h, followed by 30 min at room temperature. After discarding the medium, the cells were washed three times with PBS and crosslinked with 1 mm BS3 for 30 min at room temperature. The reaction was quenched with 20 mm Tris‐HCl (pH 8.0) for 15 minutes, and the cells were washed three times with PBS. Cells were then lysed with 700 µL IP lysis buffer, gently pipetted, and incubated on ice for 30 min. After centrifugation at 13000 rpm for 20 min, the supernatant was collected, and the protein concentration was determined using a BCA assay. 100 µg of protein was used as input, and 1 mg of total protein was used for immunoprecipitation. The IP protein was incubated with streptavidin‐coated magnetic beads at 4 °C for 4 h with continuous rotation. The peptide‐protein complex was then eluted, separated by SDS‐PAGE, and analyzed by Western blotting to detect protein interactions.

### AutoDock Protein Molecular Docking

Retrieve protein structures for FASN, BSG, CAPN2, CAV2, and DDOST from the Protein Data Bank. Conduct molecular docking using AutoDock Vina 1.1.2 software. Generate visualizations using PyMOL 2.5.4 software.

### Mutation of LACK_156‐173_ Peptide

The mutated amino acids were selected based on predictions from the ToxinPred website. The amino acids with the lowest SVM toxicity score (indicating minimal toxicity) were chosen as the mutated residues.

### Cellular Thermal Shift Assay (CETSA)

The CETSA experiment was carried out as described previously.^[^ ^36^ ^]^ MH7A cells were lysed using liquid nitrogen, and the lysates were centrifuged to collect the supernatant. Equal volumes of the samples were incubated with LACK_156‐173_ (2 mg mL^−1^) or a solvent control (DMSO) at room temperature for 1 h. The samples were then aliquoted into 50 µL per tube, heated at a specified temperature for 3 min, cooled at room temperature for 3 min, and placed on ice. After centrifugation, the supernatant (soluble fraction) was collected and analyzed by Western blotting.

### Measurement of Cellular Lipid Content

MH7A cells were stained with BODIPY 493/503 (Invitrogen, D3922) to evaluate neutral lipid levels. The fluorescence signal of BODIPY 493/503 was used as an indicator of neutral lipid levels in the cells. To perform flow cytometry (FACS) analysis, trypsinize MH7A cells and rinse them with PBS. Incubate the cells with a 2 µM BODIPY working solution in the dark at 37 °C for 15 minutes. After a quick PBS rinse, pass MH7A cells through a 70 µm filter to generate a single‐cell suspension for FACS analysis. Detect BODIPY‐positive cells using a Cytek (23‐color spectrum analyzer, USA). Analyze the data using FlowJo software. To visualize lipid droplets in MH7A cell lines using confocal imaging, culture the cells on a confocal dish (Biosharp, BS‐15‐GJM). Stain cells with 2 µm BODIPY in the dark at 37 °C for 15 min. After fixation with 4% PFA for 30 min, stain the nuclei with DAPI. Acquire images using a Nikon confocal laser scanning microscope.

### Detection of Reactive Oxygen

After trypsinization, cells were resuspended, and 1 × 10^5^ cells were incubated with 3 µm H₂DCFDA in 100 µL solution at 37 °C in the dark for 30 min. Excess dye was removed by PBS washing, and fluorescence was analyzed by flow cytometry.

### Palmitate Solution Preparation and Treatment

Palmitate (Aladdin, P101058) was dissolved in DMSO to prepare a 10 mM stock solution, aliquot, and stored at −80 °C. For palmitate rescue experiments, MH7A cells were incubated in standard FBS‐containing medium with either 200 µm palmitate in 2% BSA or 2.5‰ DMSO (as a control) for 48 h in vitro.

### Detection of FASN Activity

MH7A cells were seeded in 6 cm dishes and subjected to respective treatments, the cells were digested with trypsin and counted to obtain a density of 2 × 10^6^ cells. To evaluate the activity of FASN, a FASN activity assay kit (Solarbio) was employed following the manufacturer's instructions.

### Transfection of siRNA

Target gene siRNAs and nonsilencing control siRNAs utilized in the experiment, were obtained from RiboBio. The siRNAs target sequences are listed in Table S2 (Supporting Information). Cells were seeded in 24‐well, 12‐well, or 6‐well plates and transfected with target gene siRNA (50 nM) or negative control (NC) using Lipofectamine RNAiMAX reagent (13778150, Thermo Fisher Scientific) when cell confluence reached 70–80%. The experimental procedures were conducted 48 h post‐transfection.

### Cell Lines and Construction of FASN Knock‐Down MH7A

MH7A cells were cultured in complete RPMI 1640 medium supplemented with 10% FBS, as previously described. The PLKO.1 vector containing shRNA specifically targeting FASN was commercially procured from VectorBuilder. The shRNA targeting sequence is listed in Table S2 (Supporting Information). Subsequently, the shRNA‐FASN plasmid was transfected into HEK 293T cells along with envelope (Pmd2.G) and packaging (psPAX2) plasmids at a ratio of 4:1:3 using lipofectamine 8000 as the transfection reagent. MH7A cells were transduced with shRNA‐FASN lentivirus in the presence of polybrene and selected with 2 µg mL^−1^ puromycin for stable positive clones. Mycoplasma testing confirmed the absence of contamination in all cell lines.

### Overexpression of FASN in MH7A

A Flag‐tagged FASN overexpression plasmid was constructed and an empty vector plasmid was used as the negative control (NC). When the cells reached 60–70% confluence, transient transfection was performed using Lipo8000.

### Transmission Electron Microscopy (TEM) Analysis

RA‐FLS were harvested by trypsin digestion and fixed in 2.5% glutaraldehyde. After washing with PBS, the cells were further fixed in 1% osmium tetroxide. Following this, the cells were gradually dehydrated through a series of ethanol washes (30%, 50%, 70%, 80%, 95%, and 100%), and then infiltrated with epoxy propane, followed by a mixture of epoxy propane and epoxy resin, and finally pure epoxy resin. The samples were polymerized by heating at 40 °C for 12 h and then at 60 °C for 48 h. Ultrathin sections (70 nm) were prepared and mounted on copper grids, then stained using electron staining reagents. Imaging was performed using a JEM‐1230 transmission electron microscope.

### TOM20 Staining and Fluorescent Confocal Microscopy

MH7A were cultured with PA or transfection of small interfering RNA on a confocal dish (Biosharp, BS‐15‐GJM) for 24 h. Then, fix the cells with 4% PFA for 15 min. Block with a buffer solution (1× PBS, 5% BSA, 0.3% Triton X‐100) at room temperature for 1 h. Then, incubate the cells with the indicated primary antibody, TOM20 (1:200, CST), overnight at 4 °C, then stain the nuclei with DAPI. Capture the images using a Nikon confocal laser scanning microscope. The proportion of cells exhibiting three distinct mitochondrial morphologies was quantified as previously described.^[^ ^37^ ^]^

### Bioinformatics Analysis

Bioinformatics analysis was conducted in R statistical software (Version 4.0.2) and RStudio (Version 1.3.1073). For scRNA‐seq analysis, the primary tool used was the “Seurat” R package (version 4.0.2). The single cell expression data used was from SDY998 (https://www.immport.org/shared/study/SDY998), GSE246416, and GSE200815.^[^ ^23^ ^]^ Target genes expression analyses were visualized using Bubble diagram via “DotPlot” function.

### RNA Sequencing and Data Processing

Cell lysates were prepared using TRIzol reagent. Transcriptomics analysis was conducted using the 4 × 44K Agilent Whole Human Genome Oligo Microarray (Novogene, China) or real‐time quantitative PCR. RNA isolation followed a standard protocol as previously described.^[^ ^38^ ^]^ Align the clean sequencing data using STAR (Version 2.10.a) and perform RPKM normalization in R (Version 4.0.5). Log2‐transformed RPKM values were calculated, and gene set enrichment analysis was conducted based on the expression levels of target genes using Pheatmap (Version 1.0.12).

### Statistical Analysis

The figure legends specify the number of independent experiments and biological replicates. Data are presented as mean ± SEM, derived from at least three independent in vitro experiments. All experimental procedures, treatments, and data analyses were conducted in a blinded manner. Quantitative analysis was performed for immunoblotting and mRNA expression, with normalization to the mean value of the control group. Statistical methods included two‐tailed unpaired *t*‐tests or paired *t*‐tests for comparisons between two groups. For comparisons involving three or more groups, one‐way or two‐way ANOVA, or multiple unpaired t‐tests, were applied. A significance threshold of *p* < 0.05 was used. All statistical analyses were performed using GraphPad Prism version 10.0.

### Data Availability Statement

The raw RNA‐Seq data have been deposited in the Genome Sequence Archive (GSA: HRA010423). All code underlying the results of this study can be obtained from the corresponding author upon reasonable request.

## Conflict of Interest

The authors declare no conflict of interest.

## Author Contributions

Conceptualization, J.S.; methodology, J.S., X.F., X.L., Y.Z. S.F., and J.C.; software, J.S., S.F., Z.B., L.L., and R.H.; validation, J.S., X.F., G.F., Y.Z., S.F., Y.Y., L.Q., X.W., and Q.Z.; formal analysis, J.S., X.L., and X.F.; investigation, J.S. and X.L.; resources, J.S., G.F., L.Q., Y.Y., and X.W.; data curation, J.S.; writing—original draft preparation, J.S. and X.L.; writing—review and editing, Y.L., X.L., and Z.L., K.Y., C.G.; visualization, Z.B., R.H., L.L., and K.Y.; supervision, Y.L., X.L., and Z.L.; project administration, Y.L., X.L., and Z.L.; funding acquisition, Y.L. and X.W.

## Supporting information



Supporting Informatio

## Data Availability

The data that support the findings of this study are available from the corresponding author upon reasonable request.
