# Supplementary material for: Inhibition of Aberrant Activated Fibroblast‐Like Synoviocytes in Rheumatoid Arthritis by Leishmania Peptide via the Regulation of Fatty Acid Synthesis Metabolism
Source: Adv Sci (Weinh). 2025 Mar 24;12(19):2409154. doi: 10.1002/advs.202409154 (PMC12097062; doi:10.1002/advs.202409154)
Supplement: Supplementary file 1 — Supporting Informatio [file ADVS-12-2409154-s001.pdf]

ADVANCED SCIENCE
Open Access

Supporting Information

for *Adv. Sci.*, DOI 10.1002/advs.202409154

Inhibition of Aberrant Activated Fibroblast-Like Synoviocytes in Rheumatoid Arthritis by *Leishmania* Peptide via the Regulation of Fatty Acid Synthesis Metabolism

Jianling Su, Xuemei Fan, Yaoyao Zou, Guangtao Fu, Shiqi Feng, Xiaoxue Wang, Yongmei Yu, Lin Li, Zhenhua Bian, Rongrong Huang, Linmang Qin, Jiping Chen, Qin Zeng, Kai Yan, Caiyue Gao, Zhexiong Lian, Xin Li* and Yang Li**

Supporting Information

for *Adv. Sci.,* DOI 10.1002/advs.202409154

Inhibition of Aberrant Activated Fibroblast-Like Synoviocytes in Rheumatoid Arthritis by *Leishmania* Peptide via the Regulation of Fatty Acid Synthesis Metabolism

*Jianling Su, Xuemei Fan, Yaoyao Zou, Guangtao Fu, Shiqi Feng, Xiaoxue Wang, Yongmei Yu, Lin Li, Zhenhua Bian, Rongrong Huang, Linmang Qin, Jiping Chen, Qin Zeng, Kai Yan, Caiyue Gao, Zhexiong Lian**, Xin Li**, Yang Li**

Figure S1

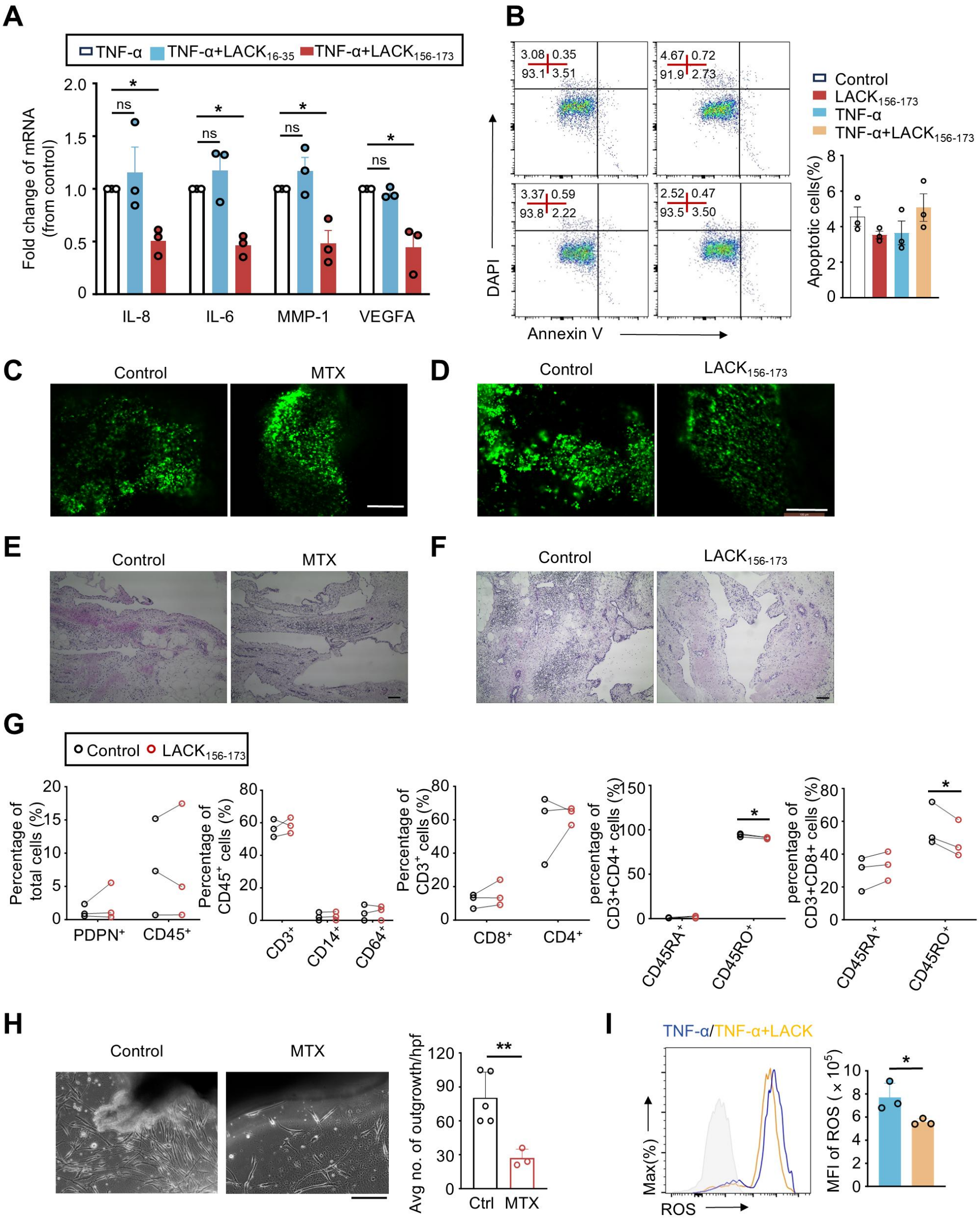

A
TNF-α
TNF-α+LACK16-35
TNF-α+LACK156-173
Fold change of mRNA (from control)
IL-8
IL-6
MMP-1
VEGFA
B
Control
LACK156-173
TNF-α
TNF-α+LACK156-173
DAPI
Annexin V
Apoptotic cells(%)
C
Control
MTX
D
Control
LACK156-173
E
Control
MTX
F
Control
LACK156-173
G
Control
LACK156-173
H
Control
MTX
Avg no. of outgrowth/hpf
Ctrl
MTX
I
TNF-α/TNF-α+LACK
Max(%)
ROS
MFI of ROS

Figure S1. Synovial Explant and RA-FLS Response to LACK Peptides and MTX. (A) qRT-PCR analyses of IL-8, IL-6, MMP-1, VEGFA expression in PBS, $LACK_{16\text{-}35}$ or $LACK_{156\text{-}173}$ in primary human RA-FLS. **(B)** Flow cytometry detection of cell apoptosis percentage, n=3. **(C, D)** The activity of synovial explants cultured for 7 days was assessed by calcein staining. Scale bar =100μm. **(E, F)** HE staining of synovial explants. **(G)** Flow cytometry analysis showed changes in the proportion of subpopulations of synovial explant cells, n=3. **(H)** Microscope (left) and statistical analysis (right) of RA-FLS outgrowths from ST explants cultured with PBS or MTX (1μM) for 1 week. The scale bar represents 100 μm (n=3-5). **(I)** Flow cytometry showed the MFI of ROS, n=3. $^*p < 0.05$, $^{**}p < 0.01$ versus vector control. The *p* values were determined by multiple unpaired t-tests (A), one-way ANOVA test (B), a paired t-test (G), and two-tailed unpaired t-test (H, I).

Figure S2

A

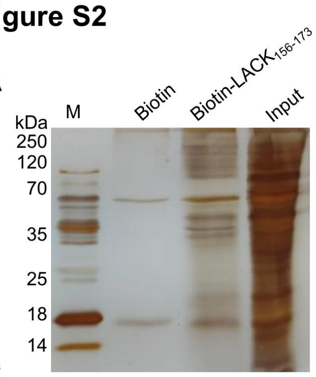

B

Subcellular location	Protein counts	Subcellular location	Protein counts
Mitochondria_nucleus	4	peroxisome	4
Mitochondria_peroxisome	2	cytosol	213
endoplasmic reticulum_Mitochondria	1	endoplasmic reticulum_Golgi apparatus	2
endoplasmic reticulum	16	Cytoskeleton_plasma membrane	6
Cytosol_peroxisome	9	plasma membrane	34
extracellular	54	cytoskeleton	3
Cytosol_Golgi apparatus	3	nucleus	160
Extracellular_plasma membrane	5	Cytosol_nucleus	180
mitochondria	48	Cytosol_mitochondria	13

C

gene	peptides	coverage%
BSG	2	12.7
CAV-2	2	20.9
CAPN2	2	3.7
DDOST	5	12.3

D

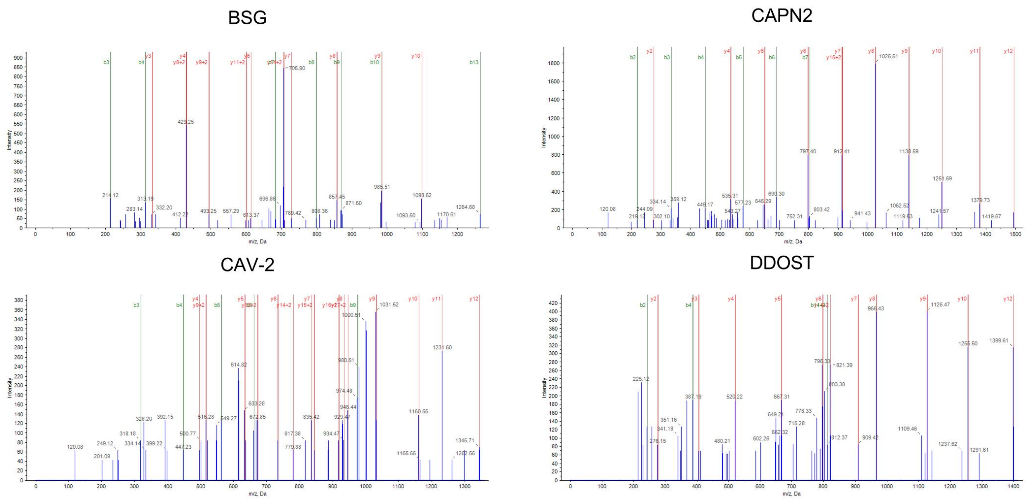

Figure S2. Proteins of RA-FLS that bind to LACK$_{156\text{-}173}$. (A) Silver staining of biotinylated LACK$_{156\text{-}173}$-associated proteins in peptide-protein pull-down assay. **(B)** Subcellular location of proteins binding to LACK$_{156\text{-}173}$ in RA-FLS, identified by mass spectrometry. **(C)** Proteomics analysis of LACK$_{156\text{-}173}$-interacted plasma membrane proteins with endocytic properties. **(D)** Secondary mass spectra of BSG, CAV2, CAPN2 and DDOST.

Figure S3

A

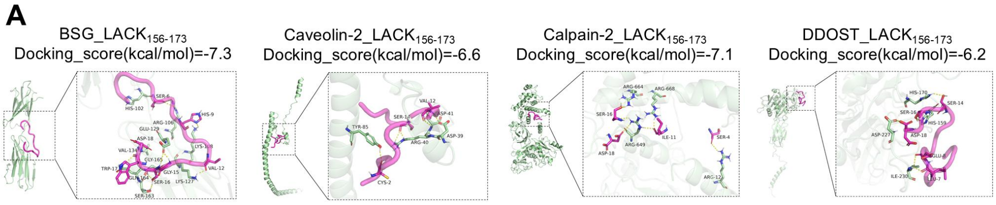

B

Peptide name	Amino acid sequence of the peptide
Biotin-LACK-WT	Biotin-Axh-ICFSPSLEHPIVVSGSWD
Biotin-LACK-Mut-BSG	Biotin-Axh-ICFSPRLEQPIYVSIVQH
Biotin-LACK-Mut-CAV2	Biotin-Axh-IYFSPSLEHPIYVWGSWD
Biotin-LACK-Mut-CAPN2	Biotin-Axh-ICFQPSLEHPRVVSGVWH
Biotin-LACK-Mut-DDOST	Biotin-Axh-ICFSPSGPHPIVVWGVWH

C **D** **E** **F**

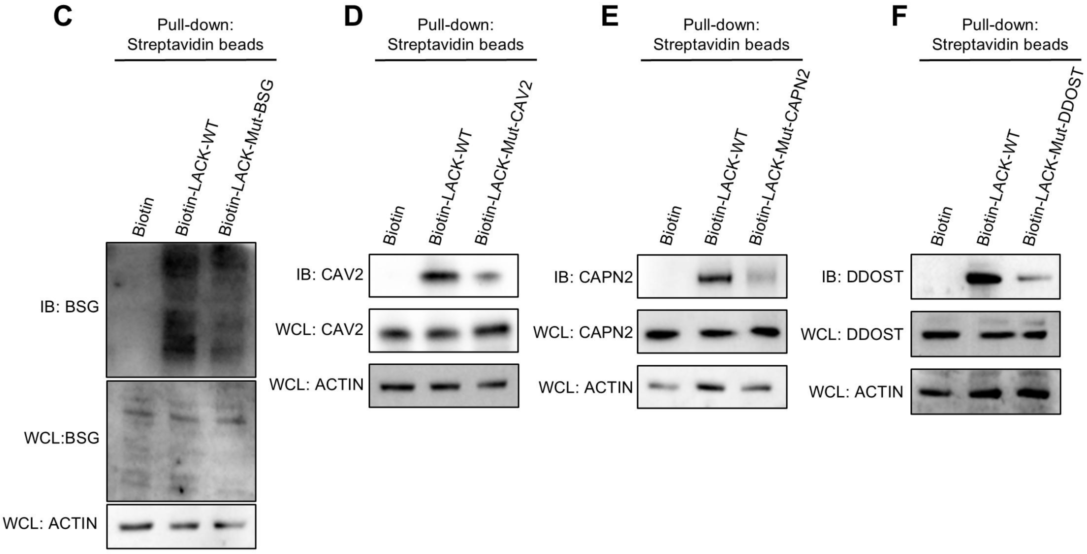

Figure S3. Mutagenesis of LACK$_{156\text{-}173}$ binding sites and their impact on protein interactions. (A) Docking models of LACK$_{156\text{-}173}$ interacting with BSG, Caveolin-2, Calpain-2, and DDOST. **(B)** Mutation sites of LACK$_{156\text{-}173}$. **(C-F)** Western blot analysis of pull-down assays assessing the interaction of mutated peptides with their respective proteins: LACK-Mut-BSG peptide with BSG protein (C), LACK-Mut-CAV2 peptide with CAV2 protein (D), LACK-Mut-CAPN2 peptide with CAPN2 protein (E), LACK-Mut-DDOST peptide with DDOST protein(F), or biotinylated WT LACK$_{156\text{-}173}$.

Figure S4

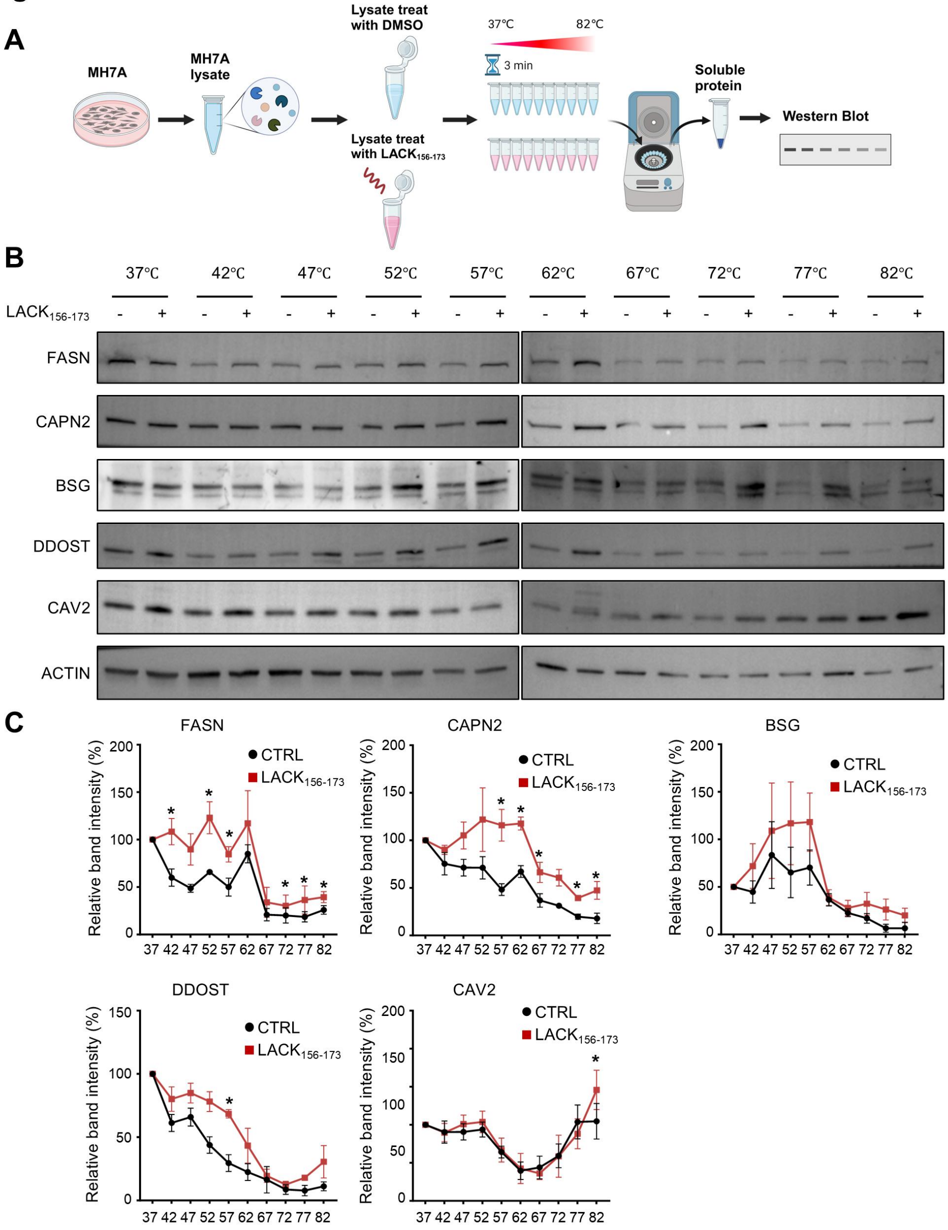

Figure S4. CETSA reveals the binding of $LACK_{156\text{-}173}$ to FASN, CAPN2, DDOST, and BSG. (A) Schematic of the CETSA-Western blot (CETSA-WB) experimental setup. Diagram is created in BioRender. Li, L. (2025) https://BioRender.com/e43b074. Agreement number: FT27VONHE6. **(B)** CETSA-WB assay demonstrating the thermal stabilization of FASN, CAPN2, BSG, DDOST, and CAV2 upon interaction with $LACK_{156\text{-}173}$ across a temperature range of 37°C to 82°C following 60 minutes of drug exposure. **(C)** Statistical analysis of the thermal stabilization data, showing significant binding of $LACK_{156\text{-}173}$ to the listed proteins. *$p < 0.05$ versus control. p-values were determined by multiple unpaired t-tests (C).

Figure S5

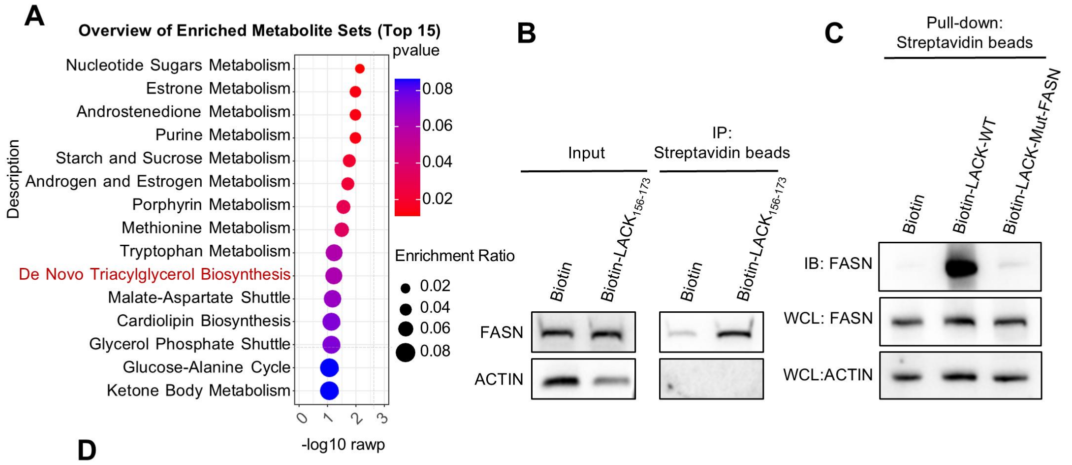

D

Peptide name	Amino acid sequence of the peptide
Biotin-LACK-WT	Biotin-Axh-ICFSPSLEHPIVVSGSWD
Biotin-LACK-Mut-FASN	Biotin-Axh-IYFSPSLEQKRVVWGVQH

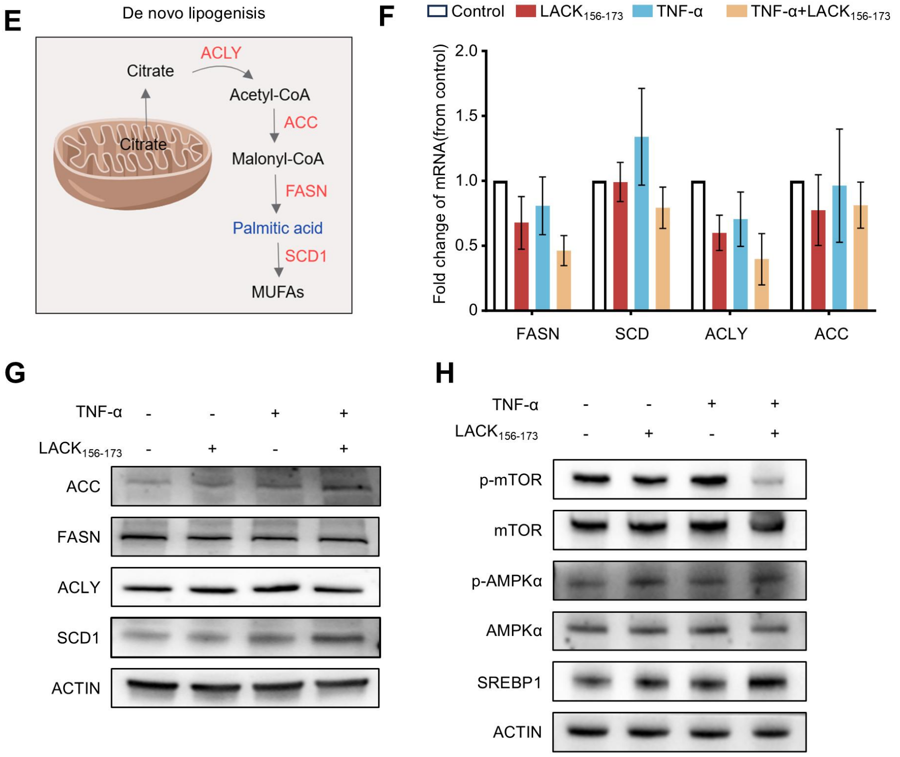

Figure S5. Impact of $LACK_{156-173}$ on fatty acid synthesis and key metabolic pathways in RA-FLSs. (A) Pathway analysis of significantly altered metabolites ($p < 0.05$, FDR < 0.05) in MH7A cells treated with $LACK_{156-173}$, following TNF-α stimulation (n=4). **(B)** Co-immunoprecipitation (CO-IP) analysis detecting the interaction between $LACK_{156-173}$ and FASN in MH7A cells. **(C)** Immunoblot analysis showing the interaction between FASN and biotinylated $LACK_{156-173}$ or -LACK-Mut-FASN peptides. **(D)** Schematic representation of the mutation site for $LACK_{156-173}$. **(E)** Overview of the de novo fatty acid synthesis pathway, highlighting key enzymes: ACLY, ACC, FASN, SCD1. **(F)** qRT-PCR analysis of mRNA expression changes in FASN, SCD1, ACLY, and ACC. **(G)** Western blot analysis assessing protein expression changes in FASN, SCD1, ACLY, and ACC. **(H)** Western blot analysis of protein expression of mTOR, AMPKα, SREBP1, and the phosphorylation of mTOR and AMPKα in RA-FLSs. The *p* values were determined by one-way ANOVA test (F).

Figure S6

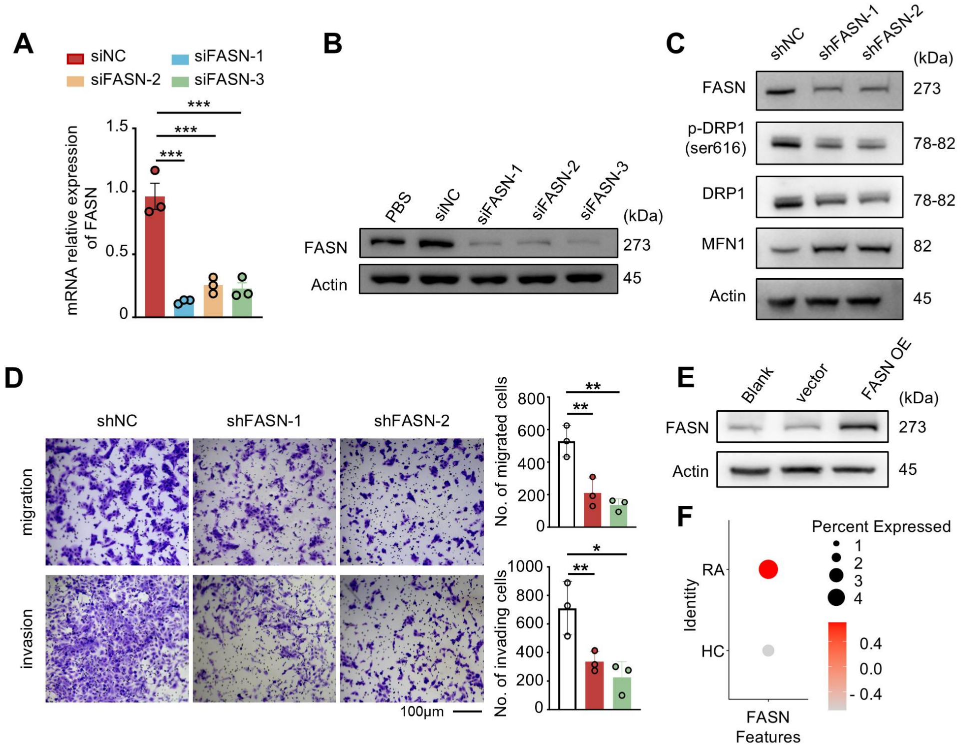

Figure S6. FASN expression and its impact on migration, invasion in MH7A cells. (A) RT-qPCR analysis of FASN expression in MH7A cells transfected with small interfering RNA (siRNA). **(B)** Western blot analysis of FASN protein levels in MH7A cells transfected with siRNA. **(C)** Western blot analysis of FASN protein expression in MH7A cells transfected with shFASN. **(D)** Transwell assay assessing the migration and invasion of MH7A cells transfected with shFASN. **(E)** Western blot analysis of FASN expression in MH7A cells overexpressing FASN. **(F)** Bubble chart shows the expression level of FASN in RA and healthy control (HC) FLS. *$p < 0.05$, **$p < 0.01$, and ***$p < 0.001$ versus vector control. The *p*-values were determined by one-way ANOVA (A, D).

Figure S7

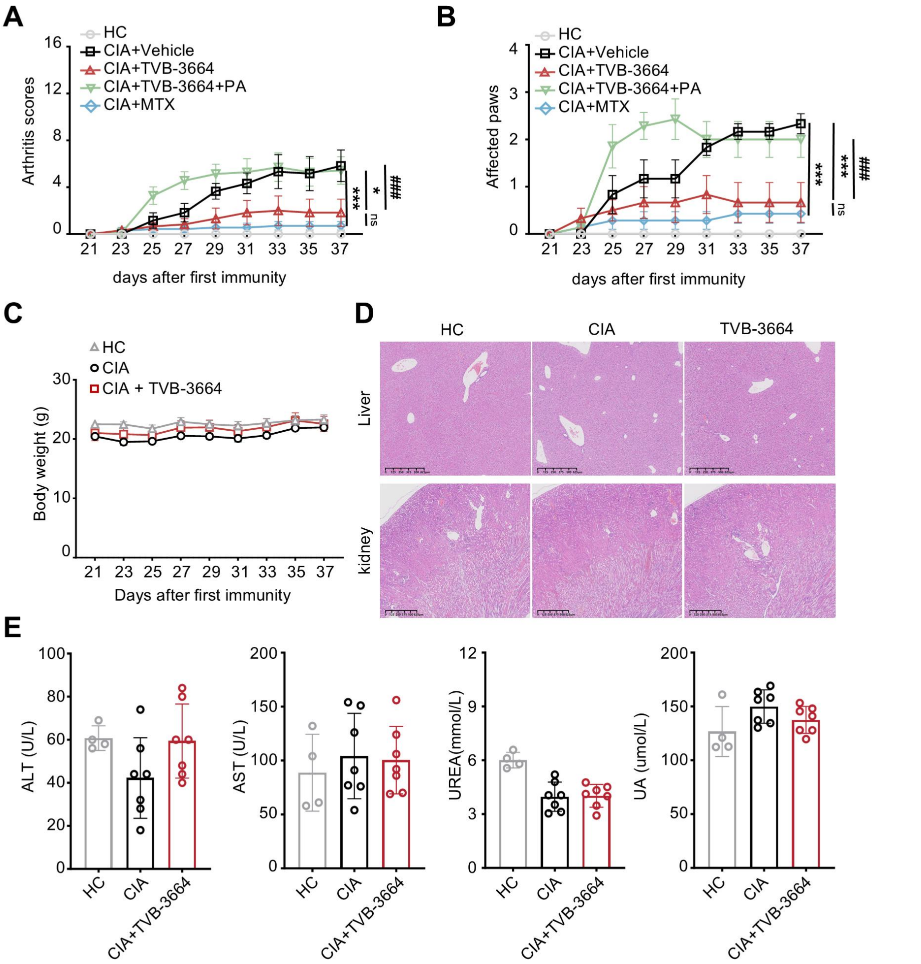

Figure S7. Evaluation of Clinical and Histological Effects of MTX and TVB-3664 Treatment in CIA Mice. (A-B) Statistical analyses of clinical scores and affected paws in CIA mice treated with TVB-3664 with or without PA, or treated with MTX (1mg/kg/week, oral gavage). ###p < 0.001, represents TVB-3664 versus TVB-3664+PA. **(C)** The statistical analyses of body weight in untreated and TVB-3664-treated mice. **(D)** Histological examination of the liver and kidney tissues after H&E staining in CIA treated with TVB-3664. Scale bar represents 625 μm. **(E)** Detection of ALT, AST, UREA, and UA in serum from HC, CIA and TVB-3664 treated CIA mice. *p < 0.05, **p < 0.01, and ***p < 0.001 versus vector control. The p values were determined by two-way ANOVA test (A-C), one-way ANOVA test (E).

Supplemental Table 1 Demographic and clinical characteristics of RA patients

Item	Patients(n=26)
Age (y)	
Range	28-79
Mean ± SD	59.29 ± 12.80
Female, No.(%)	24 (92%)
Disease duration (y)	
Median (IQR)	10 (2-15)
Tender joint count, Median(IQR)	3 (0-7)
Swollen joint count,Median (IQR)	2 (1-6)
Patient Global Assessment (0–100) , mean ± SD	49.37 ± 27.93
Physician Global Assessment (0–100), mean ± SD	40.93±23.12
DAS28-ESR, mean ± SD	4.47±1.83
C-Reactive protein (mg/L), Median (IQR)	30.70 (5.30-44.19)
IgM-rheumatoid factor-positive, No. (%)	16 (76.19%)
Anti-cyclic citrullinated peptide-positive, No. (%)	14(66.67%)
Erythrocyte sedimentation Rate, mm/hour	55.59 ± 37.73

DAS28-ESR = Disease Activity Score-28 for Rheumatoid Arthritis with Erythrocyte sedimentation Rate; IQR = interquartile range; SD = standard deviation. Categorical data are expressed as the number (%); continuous data distributed normally are expressed as the mean ± SD; continuous data distributed non-normally are expressed as the median (IQR).

Supplemental Table 2 Sequence of primers and oligonucleotides

Primers		
Human β-actin	FORWARD	CTCCATCCTGGCCTCGCTGT
	REVERSE	GCTGTCACCTTCACCGTTCC
Human IL-1β	FORWARD	TTTGAAGCTGATGGCCCTAAACA
	REVERSE	TGTAGTGGTGGTCGGAGATTCGT
Human IL-6	FORWARD	GCTGCAGGACATGACAACTCATC
	REVERSE	TCTGAGGTGCCCATGCTACATTT
Human IL-8	FORWARD	TGCTAAAGAACTTAGATGTCAGTGCAT
	REVERSE	TGGTCCACTCTCAATCACTCTCA
Human MMP-1	FORWARD	GCTAACAAATACTGGAGGTATGATG
	REVERSE	GTCATGTGCTATCATTTTGGGA
Human VEGFA	FORWARD	CAAGACAAGAAAATCCCTGTGG
	REVERSE	GCTTGTCACATCTGCAAGTACG
Human SCD	FORWARD	GCACATCAACTTCACCACATTCTTC
	REVERSE	CAGCCACTCTTGTAGTTTCCATCTC
Human ACC	FORWARD	TCTCCTCCAACCTCAACCACTATG
	REVERSE	ATTCCGCCCATCCGCTGAC
Human ACLY	FORWARD	AGACCTATGACTATGCCAAGACTATCC
	REVERSE	GCGATGCTGCCTCCAATGATG
Human FASN	FORWARD	ACAGCGGGGAATGGGTACT
	REVERSE	GACTGGTACAACGAGCGGAT
Oligonucleotides		
siFASN_001	Human	GCATCAATGTCCTGCTGAA
siFASN_002	Human	GCGTTGACCTGGTCTTGAA
siFASN_003	Human	GCATGGCTATCTTCCTGAA
shFASN-1	Human	GCTGCTAGATGTAGGTGTTAG
shFASN-2	Human	CGAGAGCACCTTTGATGACAT
siBSG	Human	TGGGCCTGGTACAAGATCA
siCAV2	Human	CAGTGCAGACAATATGGAA
siCAPN2_	Human	GAGGCCATCACGTTTCAGA
siDDOST	Human	GCTGGAGTTTGTCCGCATT
